# M6A-modified BFSP1 induces aerobic glycolysis to promote liver cancer growth and metastasis through upregulating tropomodulin 4

**DOI:** 10.1186/s43556-025-00256-9

**Published:** 2025-03-18

**Authors:** Rong Li, Shunle Li, Lin Shen, Junhui Li, Di Zhang, Jinmin Yu, Lanxuan Huang, Na Liu, Hongwei Lu, Meng Xu

**Affiliations:** 1 Department of Anesthesiology, The Second Affiliated Hospital of Xi’an JiaoTong University, Xi’an, Shaanxi, PR China; 2 Department of General Surgery, The Second Affiliated Hospital of Xi’an JiaoTong University, 157 Xiwu Road, Xi’an, Shaanxi 710004 PR China; 3 Department of Gastroenterology, The Second Affiliated Hospital of Xi’an JiaoTong University, 157 Xiwu Road, Xi’an, Shaanxi 710004 PR China; 4 Department of Oncology, The Second Affiliated Hospital of Xi’an JiaoTong University, Xi’an, Shaanxi PR China

**Keywords:** Liver cancer, m6A modification, METTL3, YTHDF1, BFSP1, TMOD4, Aerobic glycolysis

## Abstract

RNA N6-methyladenosine (m6A) is a common RNA modification in eukaryotes, and its abnormal regulation is closely related to cancer progression. Aerobic glycolysis is a main way for cancer cells to obtain energy. It was found that beaded filament structural protein 1 (BFSP1) is a m6A related gene in liver cancer. However, the effect of m6A-modified BFSP1 on aerobic glycolysis and how it is regulated in liver cancer progression have not been explored. Here, we found that BFSP1 was upregulated in liver cancer cells and tissues. Overexpression of BFSP1 promoted the viability, invasion, and aerobic glycolysis of liver cancer cells, whereas knockdown of BFSP1 showed the opposite effects. Co-immunoprecipitation, immunofluorescence and GST pull down analyses showed that BFSP1 directly interacted with tropomodalin 4 (TMOD4), and knockdown of TMOD4 reversed BFSP1 overexpression-induced malignant phenotypes and aerobic glycolysis in liver cancer cells. Moreover, methyltransferase-like 3 (METTL3) enhanced BFSP1 stability by augmenting m6A modification of BFSP1 mRNA, which is achieved in a YTHDF1-dependent manner. In vivo experiments in mice confirmed that METTL3 increased BFSP1 stability by promoting m6A modification of BFSP1 mRNA, and knockdown of BFSP1 inhibited tumor growth and metastasis. In summary, METTL3-mediated m6A methylation of BFSP1 mRNA plays an important role in the aerobic glycolysis and progression of liver cancer, providing a potential therapeutic strategy for liver cancer.

**Supplementary Information:**

The online version contains supplementary material available at 10.1186/s43556-025-00256-9.

## Introduction

Liver cancer, a primary malignant tumor of liver cells, accounts for more than 80% of primary liver cancer cases worldwide and is the second leading cause of cancer-related deaths [1]. According to related research, liver cancer is associated with alcohol consumption, viral hepatitis, diet poisons, parasites, and genetic factors [2, 3]. Liver cancer pathogenesis involves a wide range of processes, including cell cycle disorders [4], DNA methylation changes [5], N6-methyladenosine (m6A) changes [6], immune regulation [7], epithelial-mesenchymal transition [8] and increased liver cancer stem cells [9]. Although liver cancer can be treated by surgical resection, targeted therapy, immunotherapy, and liver transplantation, the treatment results are still disappointing [10, 11]. Therefore, it is of great clinical significance for early diagnosis and intervention to continue to explore the potential mechanisms of liver cancer pathogenesis and metastasis.

M6A methylation is one of the most common internal RNA modifications in eukaryotes and plays a key role in controlling RNA fate [12]. As a reversible dynamic process, m6A modification plays a pivotal role in regulating mRNA stability and structural changes, as well as RNA localization, transport, processing, and translation [13]. The m6A modification in RNA is associated with a variety of diseases, such as cardiovascular disease [14] and cancer [15]. In cancer, the disorder of m6A modification is associated with the occurrence or inhibition of tumors, which affects the malignant progression of tumors by affecting tumor proliferation, glycolysis, and apoptosis [16]. m6A RNA methylation is initiated by specific methyltransferases, named m6A ‘writers’, including methyltransferase-like 3 (METTL3), METTL14 and Wilms tumor 1-associated protein (WTAP), of which METTL3 is the key catalytic subunit [17]. Demethylation is mediated by two demethylases, m6A ‘Erasers’, alkylation repair homolog 5 (ALKBH5) and fat mass and obesity-related protein (FTO) [18]. In addition, m6A methylated RNA sites are recognized by m6A ‘readers’ (YTH domain family (YTHDFs and YTHDCs) and IGF2BP1/2/3) [19]. ‘Reader’ mainly improves RNA translation efficiency or affect RNA stability by regulating RNA degradation rate [13].

Regulators of m6A RNA methylation play an important role in tumor development. For example, METTL3 provides insights into the development and maintenance of myeloid leukemia and the self-renewal of leukemia stem cells/initiating cells through the downstream MYC pathway [20]. SUMOylation of METTL3 is important for promoting the growth of non-small cell lung cancer (NSCLC) [21]. It has also been confirmed that METTL3-mediated m6A-IGF2BP3-dependent modification upregulates LARP4B expression, thereby promoting liver cancer progression and metastasis [22]. YTHDF1 acts as a m6A reader, functioning as an oncogene or tumor suppressor in tumors [23]. It has been reported that YTHDF1 as a potential therapeutic target for hepatocellular carcinoma (HCC) promotes cancer progression [24]. Although m6A modification plays an important role in cancer, its mechanism of action and related downstream target genes in liver cancer development are largely unclear.

Beaded filament structural protein 1 (BFSP1) is a plasma membrane aquaporin 0 (AKP0/MIP)-related intermediate filament protein [25] expressed in the lens, as well as a cellular skeletal protein expressed in the lens [26]. It has been found that BFSP1 is an independent risk factor for liver cancer, and its high expression indicates a poor prognosis in patients with liver cancer [27]. BFSP1, an m6A RNA methylation-associated gene in cancer, is closely related to carbohydrate catabolism and glycolysis [28, 29]. According to a previous report, BFSP1 is a mutated gene in epithelioid gliomas, and the use of BFSP1 inhibitors may be a useful salvage treatment option for epithelioid gliomas [30]. Research has shown that tropomodalin 4 (TMOD4) binds to BFSP1 [31]. TMOD is a key effector of AMPK and plays an important role in regulating GLUT4 and glucose uptake [32]. It can be seen that TMOD4 interacts with BFSP1 and may be related to the glycolytic process. In addition, BFSP1 is associated with m6A mRNA methylation, which is associated with the survival of liver cancer patients [28]. METTL3, a key catalytic subunit of m6A modification, has been shown to play an important role in liver cancer regulation [33]. It was also found that METTL3 activates the stability of target gene transcripts in a YTHDF1 mediated m6A dependent manner [34]. Therefore, we speculated that METTL3 may mediate the m6A modification of BFSP1 mRNA and affect aerobic glycolysis in liver cancer by enhancing the stability of BFSP1 mRNA in a YTHDF1 dependent manner.

In this study, we selected BFSP1as the candidate gene, and explored the possible molecular mechanism of m6A-modified BFSP1 in the progression and metastasis of liver cancer in cell models and tumor-bearing mouse model, providing new potential therapeutic targets for liver cancer.

## Results

### BFSP1 is upregulated in liver cancer

**Fig. 1:**
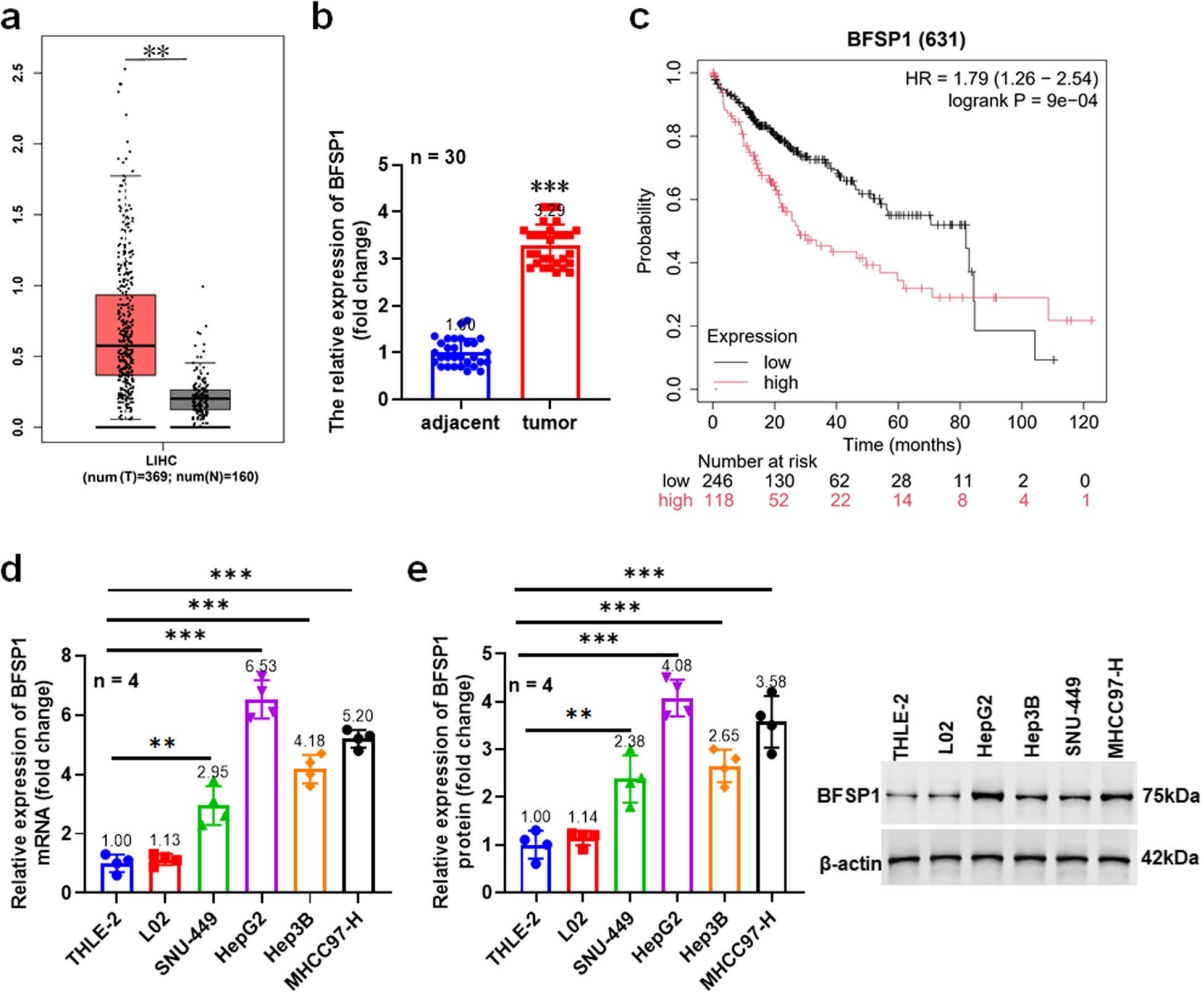
BFSP1 is upregulated in liver cancer. **a** The mRNA expression of BFSP1 in the TCGA-LIHC dataset. **b** The mRNA expression level of BFSP1 in tumor tissues (*n* = 30) and adjacent tissues (*n* = 30). **c** The relationship between the expression level of BFSP1 and the survival of liver cancer patients (*n* = 637). **d** The expression of BFSP1 mRNA (*n* = 4). **e** The expression of BFSP1 protein (*n* = 4). **P < 0.01, ***P < 0.001

To explore the role of BFSP1 in liver cancer, we first analyzed the expression levels of BFSP1 in liver cancer tissues and cells. We found that BFSP1 was upregulated in liver cancer tissues (Fig. 1a). We included 30 patients with liver cancer and detected the mRNA expression of BFSP1 in tumor tissues and adjacent tissues. The results showed that BFSP1 was upregulated in tumor tissues (Fig. 1b). In addition, the patients with high expression of BFSP1 have a poor prognosis (Fig. 1c). Quantitative real-time PCR (QPCR) and Western blotting results showed that BFSP1 was significantly upregulation in liver cancer cell lines (Fig. 1d and e).

### Knockdown and overexpression of BFSP1 affect the aerobic glycolysis of liver cancer cells

**Fig. 2:**
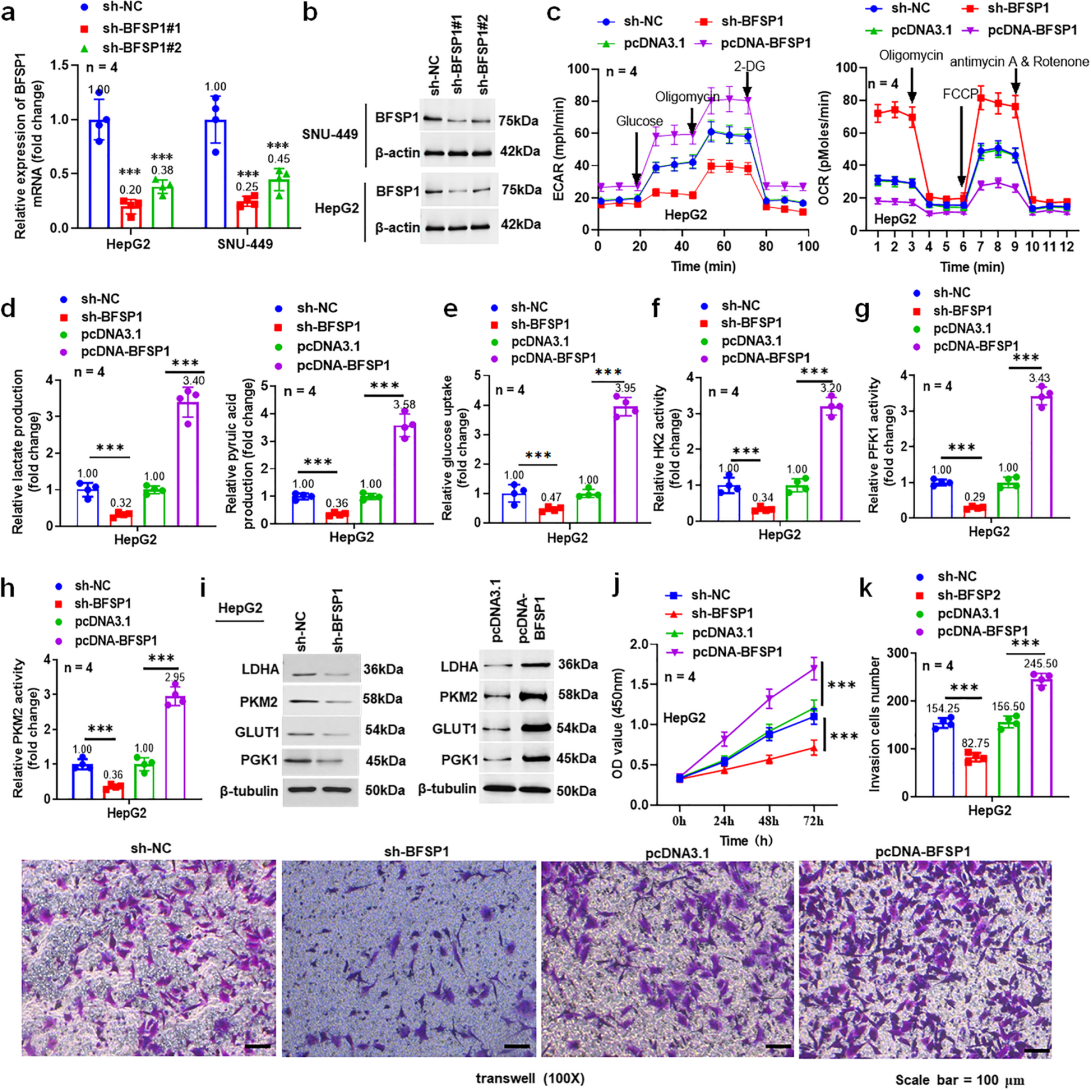
Knockdown and overexpression of BFSP1 affect the aerobic glycolysis and invasion of liver cancer cells. **a** The mRNA expression level of BFSP1 in HepG2 and SUN-449 cells (*n* = 4). **b** BFSP1 protein expression level in HepG2 and SUN-449 cells (*n* = 4). **c** The levels of ECAR and OCR in HepG2 cells. **d** The levels of lactic acid and pyruvic acid (*n* = 4). **e** Glucose uptake in HepG2 cells was detected using kit. **f–h** The activities of HK2, PFK1, and PKM2 enzymes in HepG2 cells (*n* = 4). **i** The expression levels of glycolysis-related proteins in HepG2 cells (*n* = 4). **j** The viability of HepG2 cells (*n* = 4). **k** The invasion ability of HepG2 cells (*n* = 4). ***P < 0.001

Next, we constructed sh-NC, sh-BFSP1#1, and sh-BFSP1#2 plasmids and transfected them into the HepG2 and SNU-449 cells. qPCR and western blotting showed that sh-BFSP1 significantly inhibited the expression of BFSP1 in HepG2 and SNU449 cells, and the transfection efficiency of sh-BFSP1#1 was the highest (Fig. 2a and b). Therefore, this plasmid was selected for subsequent experiments and named sh-BFSP1.

To explore the effect of BFSP1 on aerobic glycolysis of liver cancer, we transfected sh-BFSP1 and pcDNA-BFSP1 vectors into HepG2 and SNU449 cells. The result showed that knockdown of BFSP1 remarkably reduced the level of ECAR and increased the level of OCR in liver cancer cells, whereas overexpression of BFSP1 promoted ECAR level and inhibited OCR level (Fig. 2c, Fig. S1a). Knockdown of BFSP1 reduced the contents of pyruvate and lactic acid, the activities of HK2, PFK1, and PKM2, as well as the glucose uptake in liver cancer cells, whereas overexpression of BFSP1 plays the opposite role (Fig. 2d-h, Fig. S1b-f). Moreover, knockdown of BFSP1 significantly inhibited the expression of glycolysis-related proteins PKM2, GLUT1, PGK1, and LDHA in liver cancer cells, whereas overexpression of BFSP1 promoted the expression of the glycolysis-related proteins (Fig. 2i and Fig. S1g). Knockdown of BFSP1 inhibited the vitality of liver cancer cells, whereas overexpression of BFSP1 increased the vitality (Fig. 2j and Fig. S1h). In addition, knockdown of BFSP1 reduced the invasion of HepG2 cells and SNU449 cells by 46% and 45%, respectively, whereas overexpression of BFSP1 increased the invasion of HepG2 cells by 57% (Fig. 2k and Fig. S1i). The above data indicate that BFSP1 has the potential to promote the malignant phenotypes of liver cancer cells.

### BFSP1 interacts with TMOD4 and promotes their expression

**Fig. 3:**
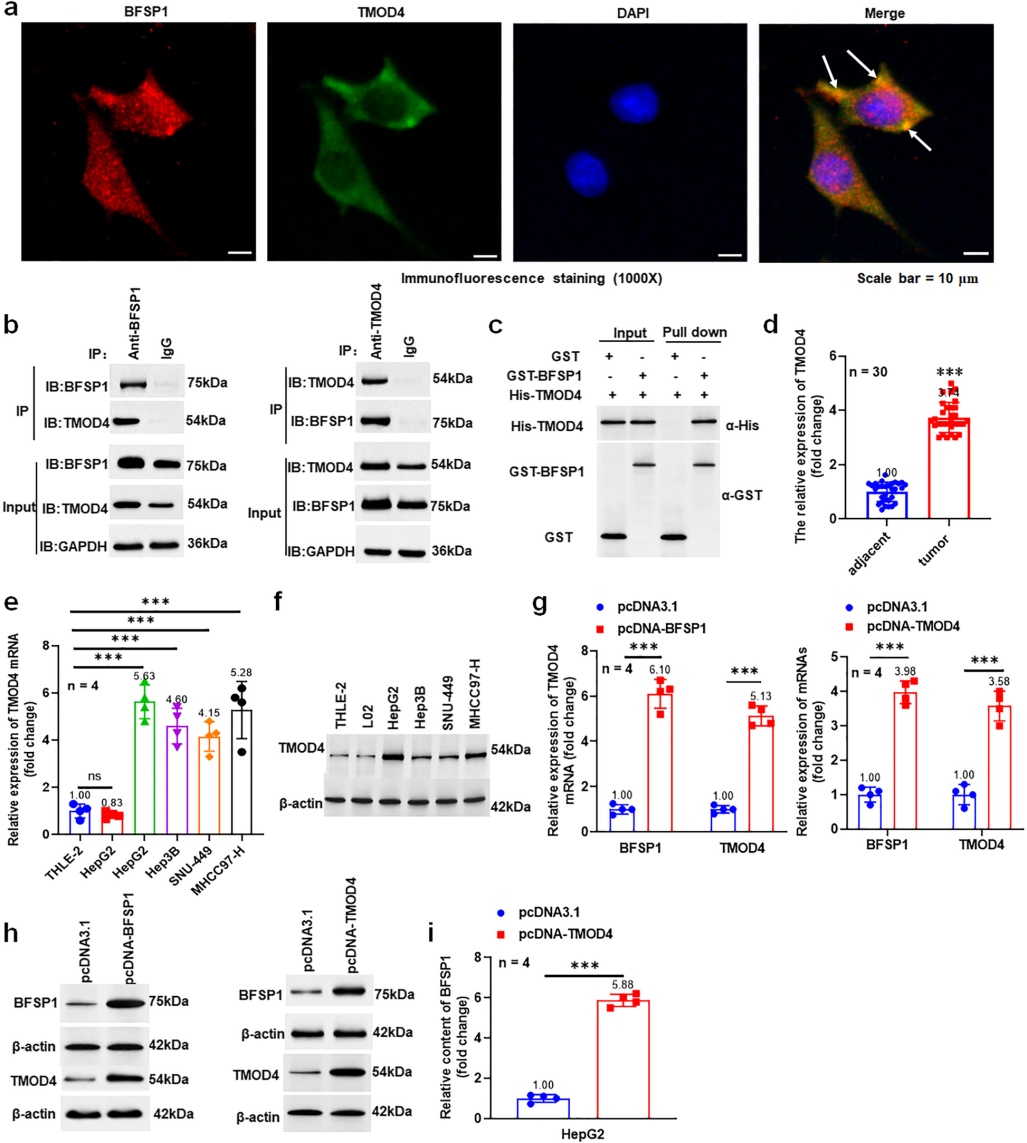
BFSP1 interacts with TMOD4 and promotes their expression. **a** Fluorescence co localization of BFSP1 and TMOD4. **b** The interaction between BFSP1 and TMOD4 was analyzed using Co-IP (*n* = 4). **c** The interaction between BFSP1 and TMOD4 was verified using GST pull down (*n* = 4). **d** The expression of TMOD4 mRNA in tumor tissues and adjacent tissues of liver cancer patients (*n* = 30). **e** The mRNA expression level of TMOD4 (*n* = 4). **f** The protein expression level of TMOD4 (*n* = 4). **g** The expression of BFSP1 and TMOD4 mRNAs in liver cancer cells (*n* = 4). **h** BFSP1 and TMOD4 protein expression levels in liver cancer cells (*n* = 4). **i** The content of BFSP1 protein in cell culture supernatant (*n* = 4). ***P < 0.001

Research has shown that TMOD4 can bind to BFSP1. Here, we mainly explored the mechanism of TMOD4 and BFSP1 in liver cancer. Immunofluorescence assay showed that BFSP1 and TMOD4 were co-localized in the cytoplasm (Fig. 3a). Co-immunoprecipitation (Co-IP) results showed that BFSP1 and TMOD4 were co-precipitated in liver cancer cells (Fig. 3b). And we also confirmed the direct binding of BFSP1 to TMOD4 by GST pull-down assay (Fig. 3c). The results showed that TMOD4 was upregulated in tumor tissues of liver cancer patients and liver cancer cell lines (Fig. 3d-f). In addition, BFSP1 overexpression promoted the expression of TMOD4 and BFSP1, and TMOD4 overexpression promoted the expression of BFSP1 and TMOD4 (Fig. 3g and h). ELISA results showed that TMOD4 also increased the content of BFSP1 in the supernatant of hepatoma cell culture (Fig. 3i). Thus, BFSP1 interacted with TMOD4, and the two promoted the expression of each other.

### TMOD4 knockdown reversed the promotion of BFSP1 overexpression on aerobic glycolysis and cell invasion of liver cancer cells

**Fig. 4:**
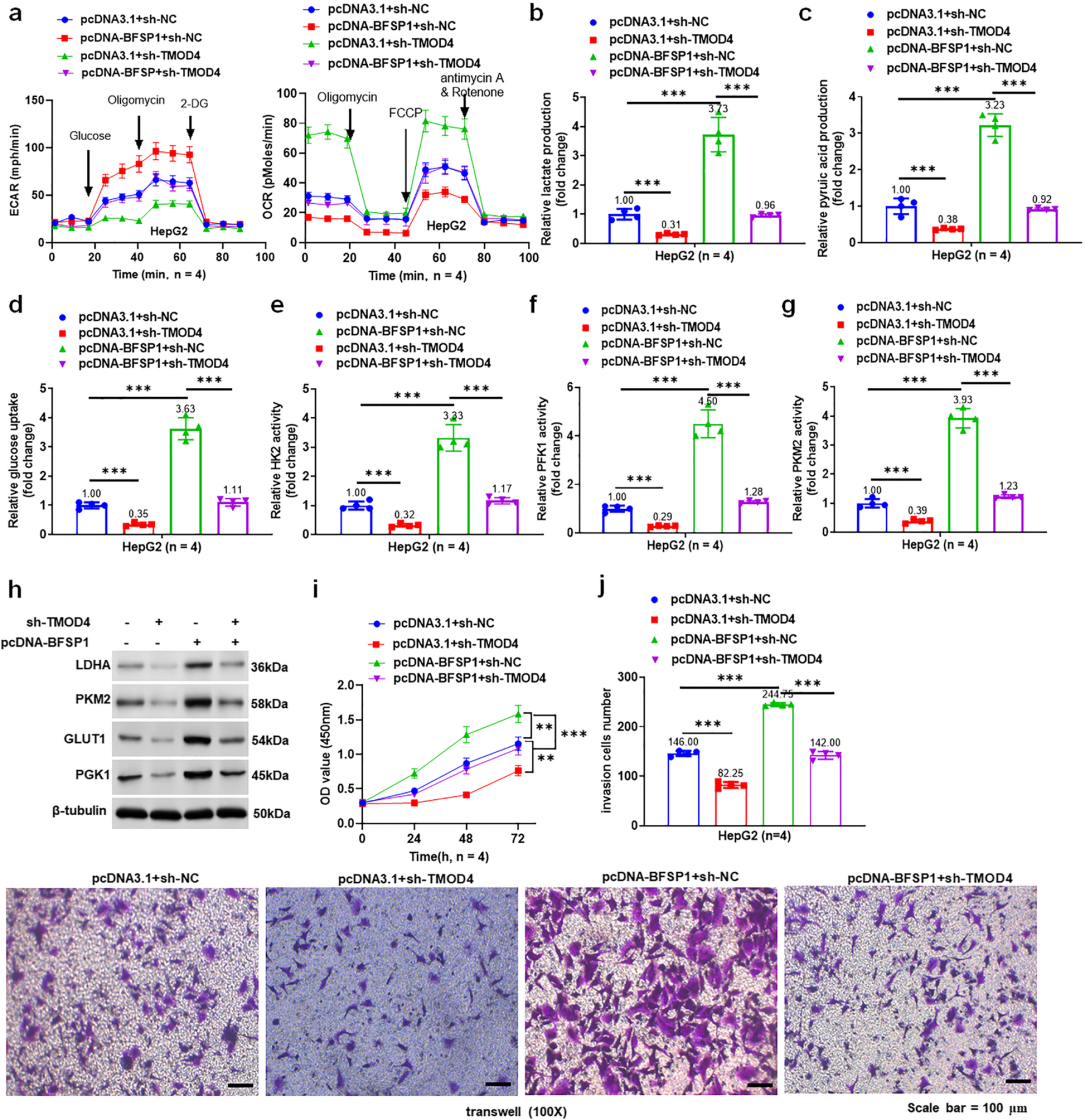
TMOD4 knockdown reversed the promotion of BFSP1 overexpression on aerobic glycolysis and cell invasion of liver cancer cells. **a** The levels of ECAR and OCR in HepG2 cells (*n* = 4). **b**, **c** The levels of lactic acid and pyruvic acid (*n* = 4). **d** Glucose uptake in HepG2 cells (*n* = 4). **e–g** The activities of HK2, PFK1, and PKM2 enzymes in HepG2 cells (*n* = 4). **h** The expression levels of glycolysis-related proteins in HepG2 cells. **i** The viability of HepG2 cells (*n* = 4). **j** The invasion ability of HepG2 cells (*n* = 4). **P < 0.01, ***P < 0.001

To explore the mechanism of BFSP1 and TMOD4 in liver cancer, pcDNA-BFSP1 and/or sh-TMOD4 were transfected into HepG2 and SNU-449 cells. The results of XFe24 cell metabolic analysis showed that pcDNA-BFSP1 increased the ECAR level and decreased the OCR level in liver cancer cells, which could be reversed by knockdown of TMOD4 (Fig. 4a, Fig. S2a). Moreover, TMOD4 knockdown restored the effects of overexpression of BFSP1 on lactic acid production, pyruvic acid production, glucose uptake, the activities of HK2, PFK1, and PKM2, and the expression of glycolysis-related proteins (Fig. 4b-h, Fig. S2b-h). The CCK8 results showed that TMOD4 knockdown offset the promotion of overexpression of BFSP1 on cell viability (Fig. 4i and Fig. S2i). The results of transwell showed that overexpression of BFSP1 increased the invasion ability of liver cancer cells by 68%, whereas knockdown of TMOD4 reversed the promotion of overexpression of BFSP1 (Fig. 4j and Fig. S2j). In summary, BFSP1 induces aerobic glycolysis and promotes invasion of liver cancer cells by interacting with TMOD4.

### METTL3 recruits YTHDF1 to upregulate BFSP1 expression via m6A

**Fig. 5:**
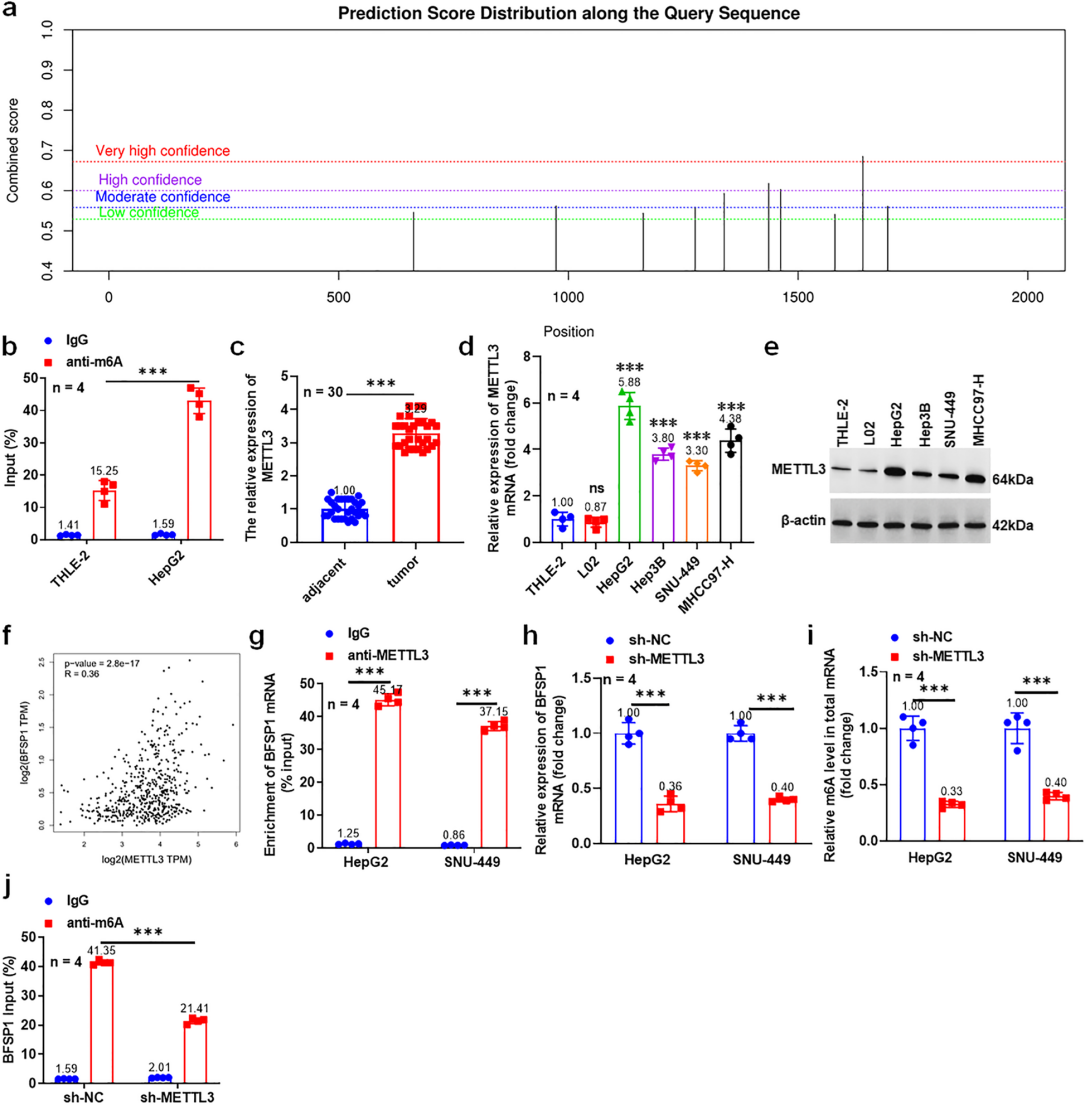
METTL3 upregulates the m6A methylation level of BFSP1. **a** BFSP1 methylation site prediction. **b** The m6A modification status of BFSP1 was determined using MeRIP-qPCR (*n* = 4). **c** The expression level of METTL3 mRNA (*n* = 30). **d** The expression level of METTL3 mRNA (*n* = 4). **e** The protein expression of METTL3 (*n* = 4). **f** Correlation analysis of METTL3 and BFSP1. **g** The direct binding of METTL3 and BFSP1 mRNA. **h** The expression level BFSP1 mRNA (*n* = 4). **i** The m6A levels of total RNA in liver cancer cells (*n* = 4). **j** The effect of sh-METTL3 on the enrichment of BFSP1 m6A modification in liver cancer cells (*n* = 4). ***P < 0.001

In order to explore whether m6A modification occurs in BFSP1 during the progression of liver cancer, SRAMP was used to predict the m6A sites of BFSP1 mRNA, and the results showed that there were multiple m6A modification sites in BFSP1 mRNA (Fig. 5a). M6-RNA Immunoprecipitation (MeRIP)-qPCR results showed that BFSP1 was significantly enriched at the m6A modification sites in liver cancer cells (Fig. 5b). Results found that METTL3 was upregulated in tumor tissues and liver cancer cell lines, and it was positively correlated with BFSP1 expression (Fig. 5c-f). RNA-binding protein immunoprecipitation (RIP) analysis showed that METTL3 binds to BFSP1 mRNA (Fig. 5g). METTL3 knockdown inhibited the expression of BFSP1 mRNA and the m6A level of total RNA in liver cancer cells (Fig. 5h and i). In addition, the enrichment of the BFSP1 in m6A modification sites was significantly reduced after knockdown of METTL3 (Fig. 5j). These results indicate that METTL3 upregulated the m6A methylation level of BFSP1mRNA in liver cancer.

**Fig. 6:**
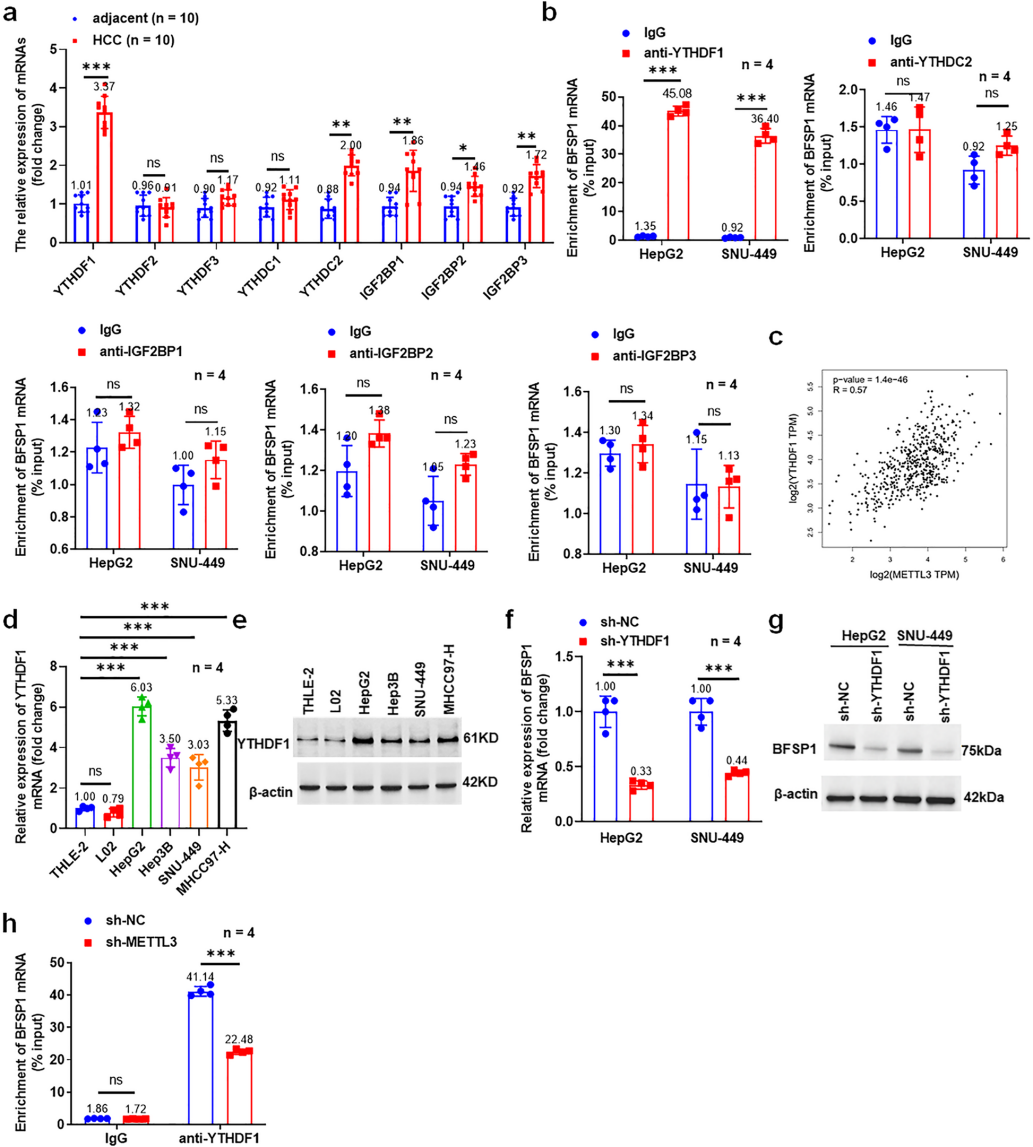
YTHDF1 is involved in m6A methylation of BFSP1 mRNA in liver cancer cells. **a** The mRNA expression levels of ‘readers’ in tumor tissues and adjacent tissues (*n* = 10). **b** Direct binding of ‘readers’ and BFSP1 mRNA (*n* = 4). **c** Correlation analysis between METTL3 and YTHDF1. **d** The expression level of YTHDF1 mRNA (*n* = 4). **e** The expression level of YTHDF1 protein in liver cancer cells (*n* = 4). **f** The expression level of BFSP1 mRNA (*n* = 4). **g** The expression level of BFSP1 protein (*n* = 4). **h** The binding enrichment of YTHDF1 and BFSP1 in METTL3 knockdown and negative control in liver cancer cells (*n* = 4). *P < 0.05, **P < 0.01, ***P < 0.001

Our results showed that YTHDF1, YTHDC2, IGF2BP1, IGF2BP1 and IGF2BP3 were significantly upregulated in liver cancer tumor tissues, of which YTHDF1 was the most significant (Fig. 6a). Subsequently, RIP-PCR results showed that only YTHDF1 bound to BFSP1 (Fig. 6b). A previous study found that METTL3 promoted the stability of target genes in a YTHDF1 mediated m6A-dependent manner [34]. Here, we found that METTL3 was significantly positively correlated with YTHDF1 in liver cancer (Fig. 6c). Therefore, we speculated that BFSP1 m6A modification induced by METTL3 could promote BFSP1 stability through YTHDF1 dependence. Moreover, YTHDF1 was upregulated in liver cancer cells, and knockdown of YTHDF1 significantly inhibited the expression of BFSP1 (Fig. 6d-g). In addition, YTHDF1 directly binds to BFSP1 mRNA, and knockdown of METTL3 reduced this binding efficiency (Fig. 6h). In summary, METTL3 induced BFSP1 m6A modification and promoted the transcriptional of BFSP1 in a YTHDF1 dependent manner.

### METTL3 enhances BFSP1 stability and promotes aerobic glycolysis and invasion of liver cancer cells in a YTHDF1 dependent manner

**Fig. 7:**
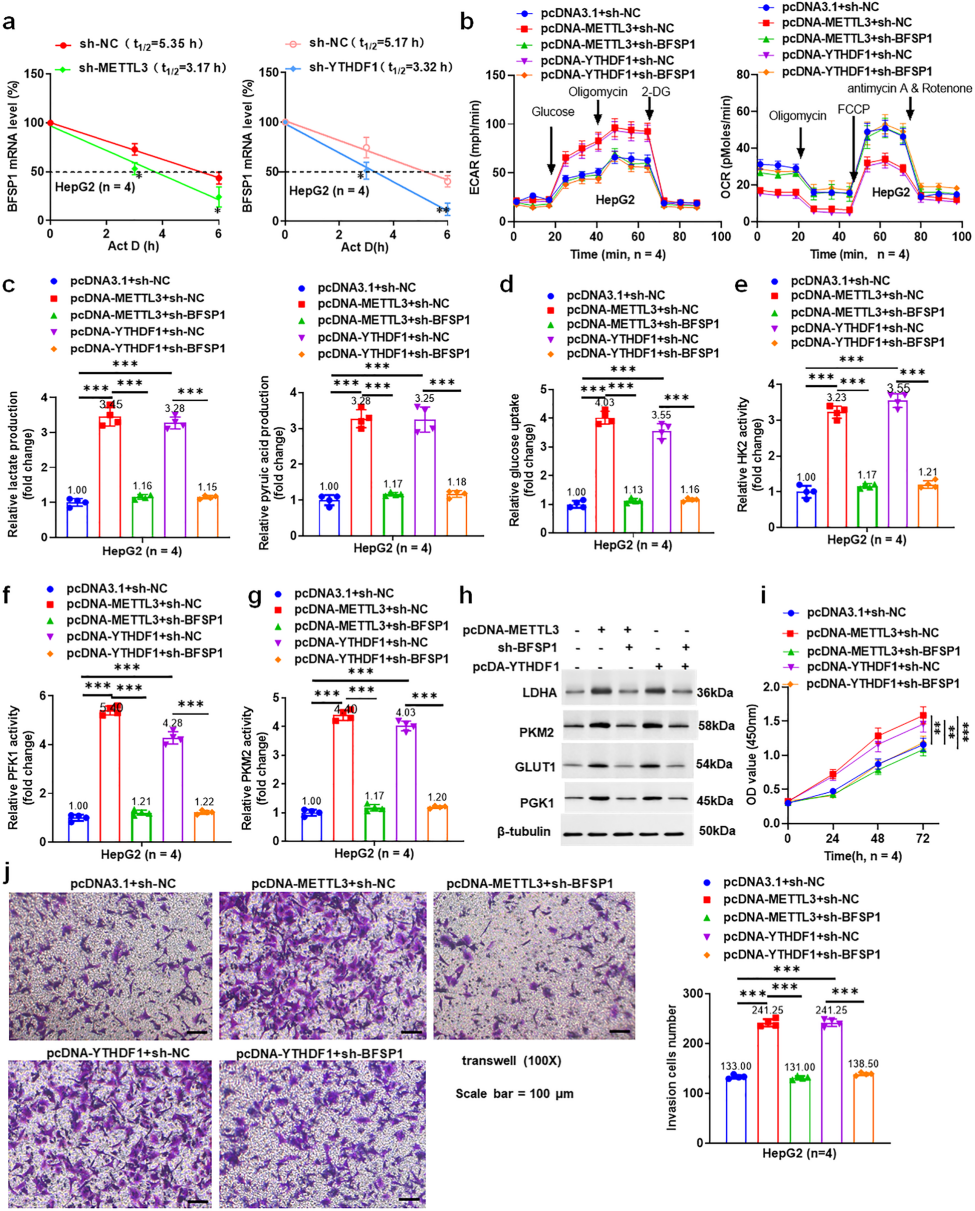
METTL3 enhances BFSP1 stability and promotes aerobic glycolysis and invasion of liver cancer cells in a YTHDF1 dependent manner. **a** The stability of BFSP1 mRNA (*n* = 4). **b** The levels of ECAR and OCR in HepG2 cells (*n* = 4). **c** The levels of lactic acid and pyruvate activity in HepG2 cells (*n* = 4). **d** Glucose uptake in HepG2 cells. **e–g** The activities of HK2, PFK1, and PKM2 enzymes. **h** The expression levels of glycolysis-related proteins in HepG2 cells (*n* = 4). **i** The viability of HepG2 cells (*n* = 4). **j** The invasion ability of HepG2 cells (*n* = 4). **P < 0.01, ***P < 0.001

The results of the BFSP1 stability assay showed that knockdown of METTL3 or YTHDF1 significantly shortened the mRNA half-life (t1/2) of BFSP1 (Fig. 7a and Fig. S3 a). The results showed that overexpression of METTL3 and YTHDF1 promoted the ECAR levels and reduced the OCR levels in liver cancer cells, which could be reversed by knockdown of BFSP1 (Fig. 7b, and Fig. S3b). Moreover, BFSP1 knockdown reversed the effects of overexpression of METTL3 or YTHDF1 on lactic acid production, pyruvic acid production, glucose uptake, the activities of HK2, PFK1, and PKM2, and the expression of glycolysis-related proteins (Fig. 7c-h, Fig. S3c-h). The results of CCK8 and transwell showed that BFSP1 knockdown offset the promotion of overexpression of METTL3 orYTHDF1 on cell viability and invasion ability (Fig. 7i, j, Fig. S3i, 3j). These results indicated that METTL3 mediated BFSP1 mRNA m6A modification enhanced BFSP1 stability and promoted aerobic glycolysis and invasion of liver cancer cells in a YTHDF1 dependent manner.

### METTL3 mediated m6A modification of BFSP1 mRNA induces viability and invasion of liver cancer cells by activating aerobic glycolysis

Then, we explored whether BFSP1 promotes liver cancer cell invasion by inducing aerobic glycolysis in liver cancer cells. The results of XFe24 cell metabolic analysis showed that 2-DG attenuated the promotion of overexpression of BFSP1 or METTL3 on ECAR level and the inhibitory effect on OCR level (Fig. S4a and S5a). Furthermore, 2-DG reversed the effects of overexpression of BFSP1 or METTL3 on lactic acid production, pyruvic acid production, cell viability and invasion ability (Fig. S4b-e, S5b-e). In summary, METTL3 mediated m6A modification of BFSP1 mRNA induced the viability and invasion of liver cancer cells by activating aerobic glycolysis.

### METTL3 promotes the progression and metastasis of liver cancer by up-regulating BFSP1 m6A level

**Fig. 8:**
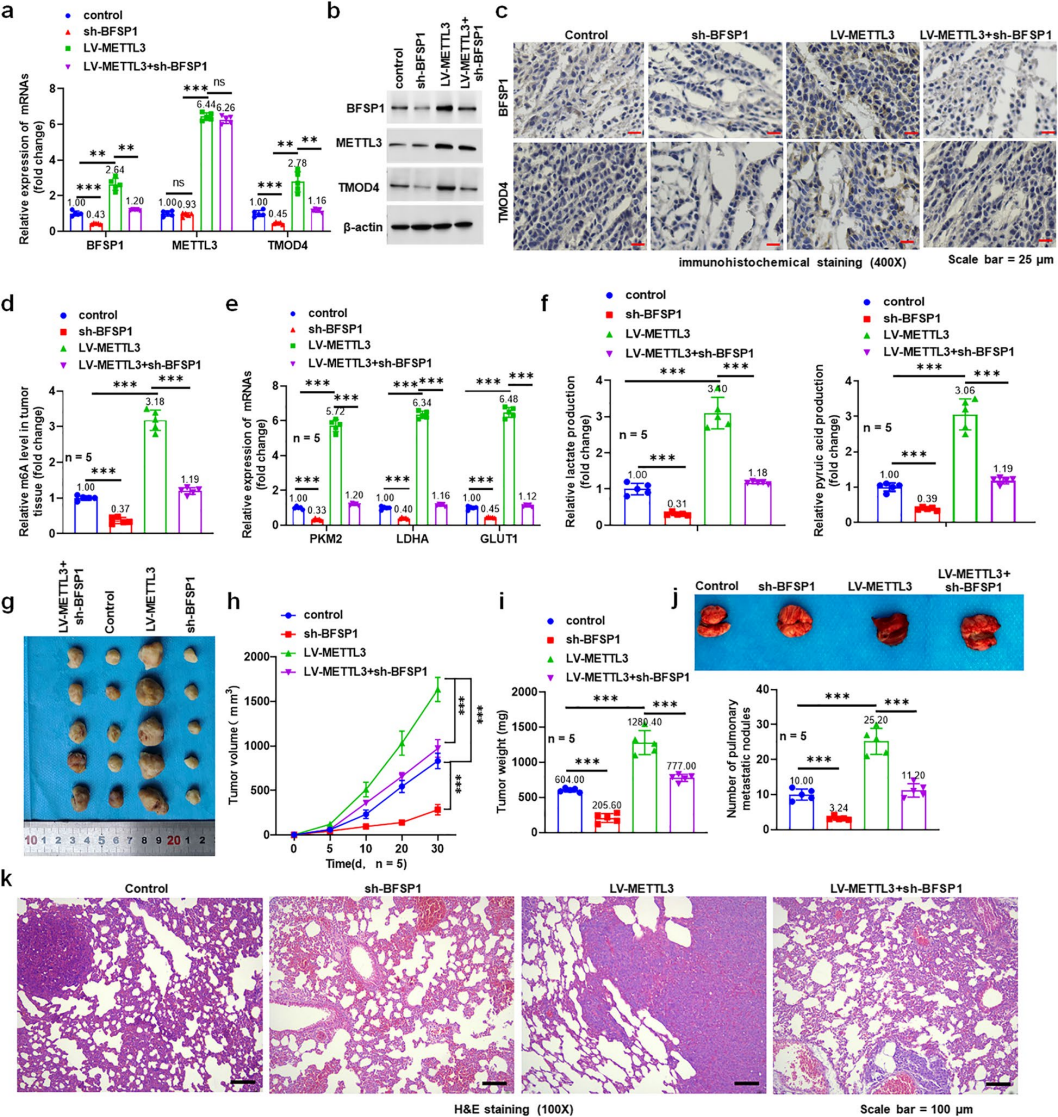
METTL3 promotes aerobic glycolysis by upregulating BFSP1 m6A level in mouse liver cancer tissues. **a** The expression levels of BFSP1, TMOD4 and METTL3 mRNA in mouse liver cancer tissues (*n* = 5). **b** The expression levels of BFSP1, TMOD4 and METTL3 proteins in mouse liver cancer tissues (*n* = 5). **c** Immunohistochemistry of BFSP1 and TMOD4 in different treatment groups. **d** The m6A level in liver cancer tissues of mice (*n* = 5). **e** The expression levels of glycolysis-related genes (PKM2, LDHA and GLUT1). **f** The levels of lactic acid and pyruvic acid in serum of mice (*n* = 5). **g** Tumor images of mice.** h** The tumor volume of mice (*n* = 5). **i **The tumor weight of mice (*n* = 5). **j** Lung tissue image of tumor lung metastasis (*n* = 5). **k** H&E staining of lung tissue sections (*n* = 5). **P < 0.01, ***P < 0.001

To investigate the effect of BFSP1 on the progression of liver cancer, the HepG2 cell lines stably overexpressing METTL3 and/or knocking down BFSP1 were injected subcutaneously into nude mice. The results showed that METTL3 overexpression promoted the expression of BFSP1, METTL3, and TMOD4 in mouse tumor tissues, whereas sh-BFSP1 reversed the effect (Fig. 8a and b). IHC results showed that METTL3 overexpression increased the protein levels of BFSP1 and TMOD4 whereas knockdown of BFSP1 offset the effects (Fig. 8c). Moreover, overexpression of METTL3 promoted the m6A levels of total RNA, which was reversed by knockdown of BFSP1 (Fig. 8d). Furthermore, overexpression of METTL3 promoted the expression of glycolysis-related genes and increased the content of lactic acid and pyruvate, which was reversed by knockdown of BFSP1 (Fig. 8e and f). After overexpression of METTL3, mice showed faster tumor growth (tumor volume increased by 96% and tumor weight increased by 2.12 times at 30 days), which was reversed by sh-BFSP1 (Fig. 8g-i). Subsequently, a mouse model of liver cancer lung metastasis was constructed. METTL3 overexpression increased the number of lung nodules, whereas knockdown of BFSP1 reversed the effect of overexpression of METTL3 on lung metastasis (Fig. 8j). H&E staining results of lung sections showed that overexpression of METTL3 promoted lung tissue damage, which was also alleviated by knockdown of BFSP1 (Fig. 8k). In summary, METTL3 accelerated the malignant progression and lung metastasis of liver cancer by upregulating BFSP1 expression.

## Discussion

Aerobic glycolysis is an important marker of cancer [35] and a key metabolic pathway in cancer cells [36]. Cancer cells usually show an enhanced dependence on glycolysis, and changes in glycolysis meet the needs of cancer cell proliferation, angiogenesis, lymph angiogenesis and metastasis [37]. The activation of aerobic glycolysis can promote the proliferation, invasion, and migration of cancer cells [38, 39]. Aerobic glycolysis can be used as an important marker for the growth, survival, and invasion of liver cancer cells [40], and induction of aerobic glycolysis promotes tumor growth in liver cancer [41]. Our study found that aerobic glycolysis occurs in liver cancer cells, and its inhibitor, 2-DG, significantly inhibits aerobic glycolysis. It has been reported that the state of m6A modification is closely related to the regulation mechanism of cancer glycolysis. According to a previous report, m6A modification of target genes affects tumor proliferation, metastasis, and treatment by regulating tumor glycolysis [42]. In addition, a study has found that m6A mRNA modification is essential for the occurrence and progression of liver cancer [43]. m6A is also involved in aerobic glycolysis in liver cancer [44]. Here, we found that METTL3-mediated BFSP1 mRNA m6A modification enhances BFSP1 stability and promotes aerobic glycolysis and invasion of liver cancer cells. It has been shown that aerobic glycolysis, as the main metabolic pathway of cancer cells, may work together with m6A modification to affect the progression of liver cancer.

Reportedly, BFSP1 is related to carbohydrate decomposition [28]. Research has found that BFSP1, as a poor prognostic marker for liver cancer, is upregulated in liver cancer [27]. Here, we found that BFSP1 was upregulated in tumor tissues of liver cancer patients and liver cancer cells. Knockdown of BFSP1 inhibited aerobic glycolysis and invasion of liver cancer cells, whereas overexpression of BFSP1 plays the opposite role. TMOD4 is a protein-coding gene. Diseases associated with TMOD4 include atherosclerosis, pulmonary hypertension, and muscle weakness [45–48]. The main function of TMOD4 is to prevent the elongation and depolymerization of actin filaments at the tip of [46, 49]. It has been reported that TMOD4 can bind to BFSP1 in a saturable manner [31]. Interestingly, TMOD4 can regulate GLUT4 and glucose uptake [32]. Bioinformatic analysis revealed that TMOD4 was significantly enriched in the glycolytic pathway [50]. In addition, TMOD4 is associated with vesicle-mediated transport, actin cytoskeleton organization, TCA cycle, and respiratory electron transport pathways. TMOD4 is also associated with glycolysis/gluconeogenesis and myocardial contraction [47]. Similar to the results of our research, we demonstrated that BFSP1 directly interacts with TMOD4 in liver cancer cells, and knockdown of TMOD4 in vitro reversed BFSP1 overexpression-induced aerobic glycolysis and invasion of liver cancer cell.

METTL3, the ‘writer’ of m6A, plays a role in tumor cell development in an m6A-dependent manner [51]. Research hasfound that METTL3 promotes the invasion and proliferation of liver cancer cells by inducing m6A modification of target genes [6, 52]. This study found that METTL3 was upregulated in liver cancer and facilitated the malignant phenotype of cancer cells by promoting m6A methylation of BFSP1 mRNA. Moreover, METTL3 mediates the expression of REG1α in an m6A dependent manner to activate glycolysis process of colorectal cancer cells [53]. METTL3 upregulates m6A modification of adenomatous polyposis coli by recruiting YTHDF, resulting in aerobic glycolysis in mice [54]. Significantly, METTL3 also enhances the stability of HK2 mRNA through YTHDF1 mediated m6A modification, thereby promoting aerobic glycolysis in cervical cancer [34]. In addition, METTL3 drives aerobic glycolysis in liver cancer cells by mediating target gene m6A modifications [55]. This finding is consistent with the results of the present study. Here, we found that METTL3 induces aerobic glycolysis in liver cancer by upregulating m6A modification of BFSP1, which can be achieved by recruiting YTHDF1.

Tumor metastasis is a process in which tumor cells escape from the primary site, spread through lymphatic/blood circulation, and eventually spread to distant sites. It is the main cause of death in cancer patients [56]. Research has found that overexpression of METTL3 promotes liver metastasis of gastric cancer (GC) [57]. METTL3 mediated YAP1 m6A modification promotes liver metastasis of lung adenocarcinoma [58]. METTL3 also promotes lung metastasis of GC [59]. In liver cancer, METTL3 promotes the expression of SOCS2 through YTHDF2 dependent enhancement of SOCS2 m6A modification, which in turn induces lung metastasis of liver cancer [60]. In addition, METTL3 promotes lung metastasis of liver cancer by promoting GBAP1 m6A modification to induce GBAP1 expression [52]. METTL3 can also catalyze YTHDF1 mediated m6A to increase Snail mRNA translation and epithelial-mesenchymal transition to induce liver cancer metastasis [61]. In this study, our in vivo experiments found that METTL3 induced the growth of mouse liver cancer tumors and induced lung metastasis of tumors by upregulating the m6A methylation level of BFSP1 mRNA, which was similar to the results of previous studies. Thus, targeting the METTL3-YTHDF1-BFSP1 axis may be a potential strategy for liver cancer treatment.

The current research still has limitations. First, although we have confirmed the role of METTL3/BFSP1/TMOD4 axis in the progression and metastasis of liver cancer, since this study is only verified in cell and liver cancer xenograft models, other animal models are needed for further verification. On the other hand, whether the BFSP1/TMOD4 axis can be used as a clinical indicator of liver cancer needs to be further verified by expanding clinical samples. In addition, tumor metastasis is a multi-stage complex process. This study demonstrates the effect of BFSP1 on lung metastasis of liver cancer, and the effect of BFSP1 on the metastasis pathway and mode of liver cancer in vivo needs to be further explored. Nevertheless, this study still provides us with a new perspective to reveal the molecular mechanism of BFSP1 regulating liver cancer progression and metastasis. These findings provide new insights for future research on liver cancer.

In summary, we found that BFSP1 was upregulated in liver cancer, and BFSP1 and its interacting protein TMOD4 work together to affect the viability, invasion, and aerobic glycolysis of liver cancer cells. Additionally, METTL3 enhances the stability of BFSP1 mRNA in a YTHDF1 dependent manner, thereby promoting aerobic glycolysis in liver cancer cells. In vivo experiments showed that METTL3 induced the growth of mouse liver cancer tumors by upregulating m6A methylation of BFSP1. This study expands our understanding of the m6A modification of BFSP1 mRNA in liver cancer, indicating that BFSP1 may have potential value**s** in the treatment of liver cancer.

## Materials and methods

### Collection of liver cancer patients

Thirty patients with liver cancer admitted to the Second Affiliated Hospital of Xi’an JiaoTong University were selected. The eligibility criteria for the recruitment of liver cancer patients are as follows: histologically confirmed liver cancer patients, surgical resection, complete clinical and follow-up data, no anti-cancer treatment before surgery, no history of other malignant tumors, and survival at least 1 month after surgery. The study was approved by the Ethics Committee of Xi’an JiaoTong University (Approval number: XJTU-2023-76H).

### Cell culture

Human liver immortalized cell line THLE-2 was purchased from Yuchi Biotechnology (SC0659, Shanghai, China). Liver cancer cell lines (HepG2 (CC0101), Hep3B (CC0103), SNU-449 (CC0105), and MHCC97-H (CC0109)) were purchased from the Guangzhou CELLCOOK (China). All cells have undergone STR identification. The cell lines were cultured in DMEM medium containing dual antibodies (HyClone, Logan, UT, USA) at 37 °C in an incubator containing 5% CO_2_.

### Cell transfection

sh-METTL3, sh-YTHDF1, sh-BFSP1, and sh-TMOD4 were designed and constructed by GenePharma (Shanghai, China). pcDNA-BFSP1, pcDNA-METTL3, pcDNA-YTHDF1, and pcDNA3.1, were purchased from Thermo Fisher Scientific. Cells were digested with trypsin, and the single cell suspension was inoculated in a cell culture plate and cultured overnight in a 37 °C, 5% CO_2_ incubator. The above vectors were transfected into liver cancer cells using Lipofectamine 2000 Reagent (Thermo Fisher Scientific).

The lentiviral overexpression vector (LV-METTL3), shRNA lentiviral plasmid (sh-BFSP1), and empty control were constructed by Invitrogen Co., Ltd. (USA). Subsequently, sh-BFSP1 and METTL3 were introduced into liver cancer cells using a lentivirus system.

### QPCR

Total RNA was isolated using TRIzol reagent (Thermo Fisher Scientific). Subsequently, qPCR was performed according to the method reported by Zhang et al. [62]. Quantification was performed using the 2^−ΔΔCt^ method after normalization to an internal control (β-actin). The primers used for qPCR were shown in Supplementary Table 1.

### Western blotting

A protein extraction kit (Thermo Fisher Scientific) was used to isolate total protein. The obtained proteins were quantified by the BCA protein assay kit (Thermo Fisher Scientific). Subsequently, Western blot analysis was performed according to the method reported by Zhang et al. [63]. The antibodies used are shown in Supplementary Table 2.

### Co-IP analysis

A total of 5 × 10^6^ liver cancer cells were collected and centrifuged at 4 °C and 1000 rpm. Cells were lysed with a pre-cooled mixture containing protease inhibitors and centrifuged. Subsequently, IgG antibody and protein A/G beads are added and incubated for 2 h at 4 °C in a shaker. After removal of the beads, centrifuge and add an appropriate amount of anti-BFSP1 or anti-TMOD4 and shake slowly at 4 °C overnight. Then, magnetic beads were added to bind to the antigen–antibody complex. Protein binding was detected by Western blot analysis.

### Immunofluorescence staining

Liver cancer cells were fixed with 4% paraformaldehyde for 20 min, and the cells were treated with 0.1% Triton X-100. The samples were covered with 2% BSA PBST blocking solution and incubated at room temperature for 1 hour. After removing the blocking solution, the sample was added with primary antibody and incubated at room temperature for 2 hours. The secondary antibody labeled with fluorescein was added dropwise and incubated at room temperature in dark for 1 hour. Finally, the nucleus was stained with DAPI-PBS. ImageJ software was used to analyze the images.

### ECAR and OCR analysis

Prepare the test solution using Seahorse XFBase Medium and add the desired substrate. Liver cancer cells in logarithmic growth phase were collected and seeded into Seahorse XF24 cell culture plates (Seahorse Bioscience, MA, USA) with 4 × 10^4^ cells per well. After the cells were adherent, the growth medium was replaced with a test solution, and then the cells were placed in a 37 °C CO_2_-free incubator for 1 h. Subsequently, the cell culture plate was placed in the Seahorse XFe24 cell metabolism analyzer to detect ECAR and OCR.

### Detection of glycolysis products and activity of key enzymes

Lactic acid and pyruvate were detected using a Lactate Assay Kit (MAK064, Sigma-Aldrich, USA) and a Pyruvate Assay Kit (MAK071, Sigma-Aldrich). HK2 ELISA kit (BLL-S7455G, Baililai Biology, China) was used to detect HK2 activity, a PFK1 ELISA kit (MY04483, Enzymes Biotechnology, China) was used to detect PFK1 activity, a PKM2 ELISA detection kit (YT-H12135, Yueteng Biotechnology, China) was used to detect PKM2 activity, and a BFSP1 ELISA kit (KPR-H21329, Kepeirui Biotechnology, China) was used to detect BFSP1 concentration.

### Glucose uptake detection

A Glucose Uptake Assay kit (ab136955, Abcam) was used to detect glucose uptake during aerobic glycolysis. In short, different treatments of liver cancer cells were inoculated into different 96-well plates and cultured overnight. Glucose-free medium was added and cultured at 37 °C for 15 min. Next, a Probe solution diluted 500 times with glucose-free medium was added and cultured at 37 °C for 15 min. After washing three times with WI Solution, which was pre-cooled to 4 °C, WI Solution was added again. Finally, the absorbance at 520 nm was detected by fluorescence microplate reader.

### SRAMP

The sequence of the BFSP1 gene was input into the SRAMP online tool, and the Mature mRNA mode was selected and submitted to predict the m6A modification site on the BFSP1 RNA sequence.

### m6A RNA methylation quantification

The m6A level of total RNA was detected using an m6A RNA methylation assay kit (ab185912, Abcam). Briefly, the corresponding solution was added to each well and incubated at 37 °C for 90 min, after which m6A capture antibody and detection antibody were added. Subsequently, 100 μL of termination solution was added to terminate the enzyme reaction. The absorbance was measured at 450 nm to evaluate the m6A level.

### MeRIP qPCR Assay

RNA was extracted from cells and mouse tumor tissues, respectively. The levels of m6A were analyzed according to the instructions of Magna MeRIP TM m6A kit (17–10499, Billerica, MA, USA).

### RIP assay

A Magna RIP kit (17–700, Merck Millipore, Germany) was applied to conduct the RIP assay. In short, liver cancer cells (2 × 10^7^) were lysed using RIPA. Preparation of protein A/G and antibody complexes. Subsequently, the cell lysate was added to the complex and incubated overnight at 4 °C. RNA was extracted after purification and qPCR analysis was performed.

### RNA stability analysis

0.2 mM actinomycin D (Act D) was used to treat the liver cancer cells (53600ES08, Yisheng Biotechnology, China) for 0, 3, and 6 h. Cells were collected, RNA was extracted using TRIzol reagent, and mRNA expression was determined by qPCR.

### Xenograft tumor assay

BALB/c nude mice (4–6-week-old, 18–22 g) were purchased from the Animal Experiment Center of the Xi’an JiaoTong University. All mice were housed under pathogen-free conditions with continuous access to food and water. The mice were randomly divided into control, sh-BFSP1, METTL3, and METTL3 + sh-BFSP1 group. Liver cancer cells transfected with lentiviral vector (1.0 × 10^10^ cells/0.10 mL PBS) were subcutaneously injected into nude mice. The mice were sacrificed and the tumor tissues were separated and weighed after 30 days of modeling. This study was approved by the Animal Care and Use Committee of the Xi’an JiaoTong University (Approval number: XJTULAC-2022-042 J).

### H&E staining

The lung tissue of mice was removed and fixed with 10% formalin solution to make 4 μm tissue sections. After dewaxing, the tissue sections were stained with hematoxylin for 10 min, and re-stained with 1% eosin for 1 min. After washing with deionized water, the samples were dehydrated with gradient ethanol, transparent with xylene, and fixed with neutral gum. Tissue lesions were observed and photographed using an optical microscope.

### Statistical analyses

GraphPad 8.0.2 and SPSS 22.0 were used for data analyses. Student’s t-test and ANOVA were used to analyze the significant difference. All data were expressed as mean ± SD. *P* < 0.05 was considered significant.

## Supplementary Information

Supplementary Material 1.

## Data Availability

The datasets generated in the current study are available from the corresponding author upon reasonable request.
