# Supplementary material for: M6A-modified BFSP1 induces aerobic glycolysis to promote liver cancer growth and metastasis through upregulating tropomodulin 4
Source: Mol Biomed. 2025 Mar 18;6:17. doi: 10.1186/s43556-025-00256-9 (PMC11914548; doi:10.1186/s43556-025-00256-9)

**M6A-modified BFSP1 induces aerobic glycolysis to promote liver cancer growth and metastasis through upregulating TMOD4**

**Running title:** BFSP1 enhances liver cancer aerobic glycolysis

Rong Li ^1, #^, Shunle Li ^2, #^, Lin Shen ^3^, Junhui Li ^2^, Di Zhang ^2^, Jinmin Yu ^3^, Lanxuan Huang ^4^, Na Liu ^3,^ *, Hongwei Lu ^2,^ *, Meng Xu ^2,^ *

^1^Department of Anesthesiology, The Second Affiliated Hospital of Xi’an JiaoTong University, Xi’an, Shaanxi, PR China; ^2^Department of General Surgery, The Second Affiliated Hospital of Xi’an JiaoTong University, Xi’an, Shaanxi, PR China; ^3^Department of Gastroenterology, The Second Affiliated Hospital of Xi’an JiaoTong University, Xi’an, Shaanxi, PR China; ^4^Department of Oncology, The Second Affiliated Hospital of Xi’an JiaoTong University, Xi’an, Shaanxi, PR China.

^#^ These authors contributed equally to this study.

*Corresponding authors:

Dr. Na Liu, Department of Gastroenterology, The Second Affiliated Hospital of Xi’an JiaoTong University, 157 Xiwu Road, Xi’an 710004, PR China, Email: Liunafmmu@163.com

Dr. Hongwei Lu, Department General Surgery, The Second Affiliated Hospital of Xi’an JiaoTong University, 157 Xiwu Road, Xi’an 710004, PR China, Email: lhwdoc@163.com

Dr. Meng Xu, Department General Surgery, The Second Affiliated Hospital of Xi’an JiaoTong University, 157 Xiwu Road, Xi’an 710004, PR China, E-mail: miaoslnol041135@163.com; xm19912015@163.com

**Supplementary figures**

**
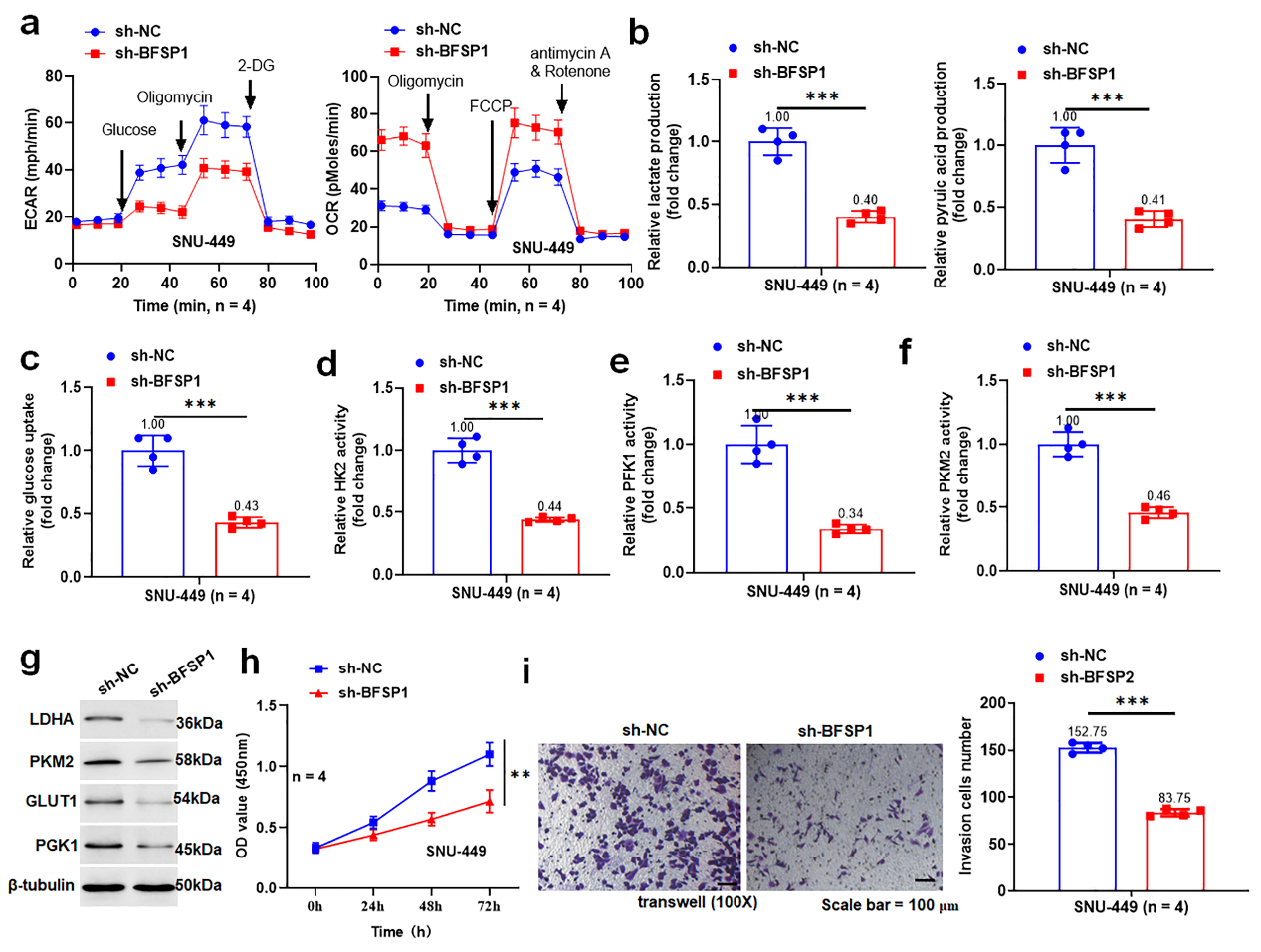
**

**Fig. S1 Knockdown of BFSP1 inhibited aerobic glycolysis and invasion of SNU-449 cells**

**a.** The levels of ECAR and OCR in SNU-449 cells (n = 4). **b.** The levels of lactic acid and pyruvate in SNU-449 cells (n = 4). **c.** Glucose uptake in SNU-449 cells (n = 4). **d-f.** The activities of HK2, PFK1, and PKM2 enzymes in SNU-449 cells (n = 4). **g.** The expression levels of glycolysis-related proteins in SNU-449 cells (n = 4). **h.** The viability of SNU-449 cells (n = 4). **i.** The invasion ability of SNU-449 cells (n = 4). ***P* < 0.01, ****P* < 0.001.

**
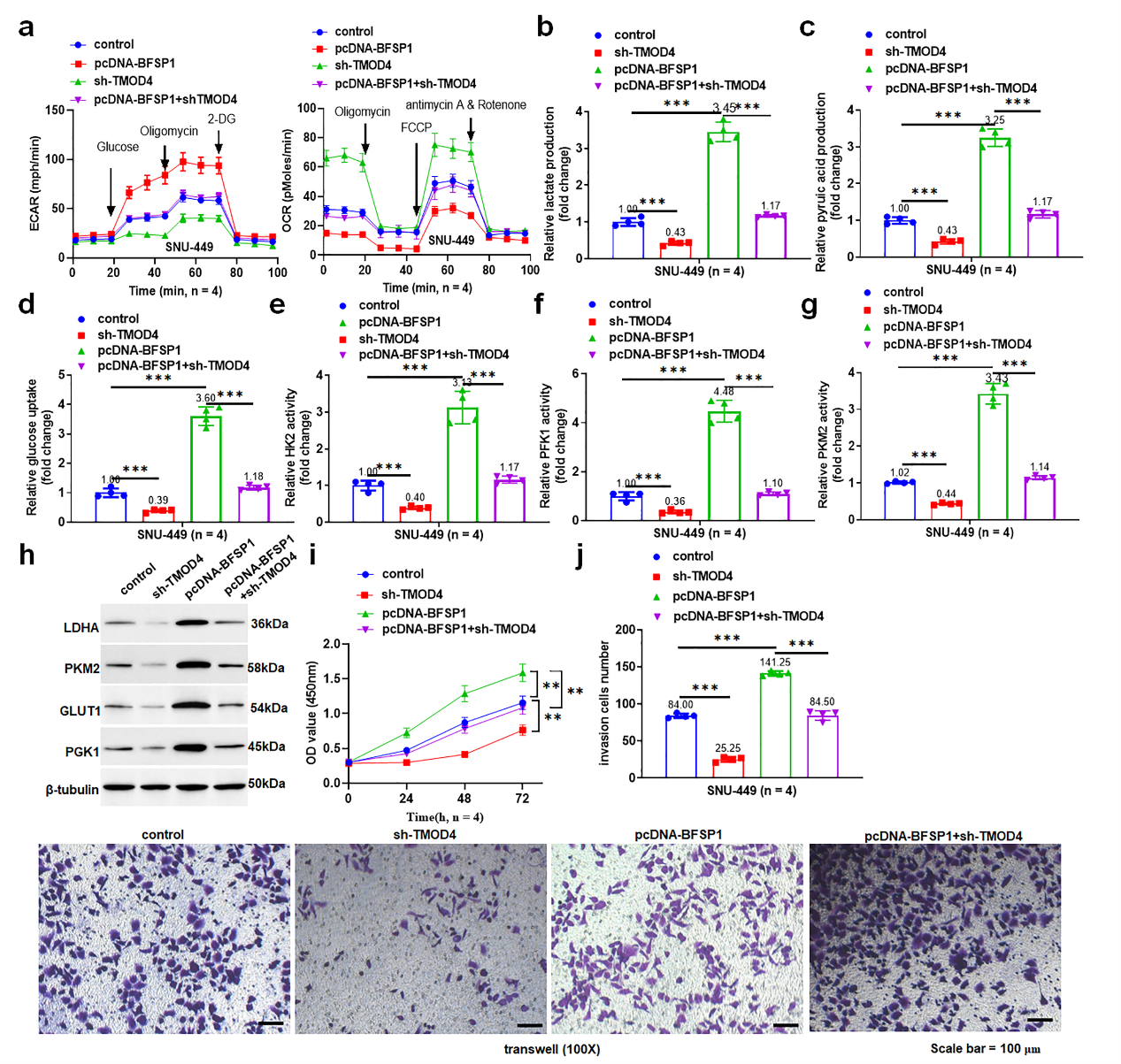
**

**Fig. S2 Knockdown of TMOD4 reversed the effects of BFSP1 on aerobic glycolysis and invasion of SNU-449 cells**

**a.** The levels of ECAR and OCR in SNU-449 cells (n = 4). **b-c.** The levels of lactic acid and pyruvate n SNU-449 cells (n = 4). **d.** Glucose uptake in SNU-449 cells (n = 4). **e-g.** The activities of HK2, PFK1, and PKM2 enzymes in SNU-449 cells (n = 4). **h.** The expression levels of glycolysis-related proteins in SNU-449 cells (n = 4). **i.** The viability of SNU-449 cells (n = 4). **j.** The invasion ability of SNU-449 cells (n = 4). ***P* < 0.01, ****P* < 0.001.


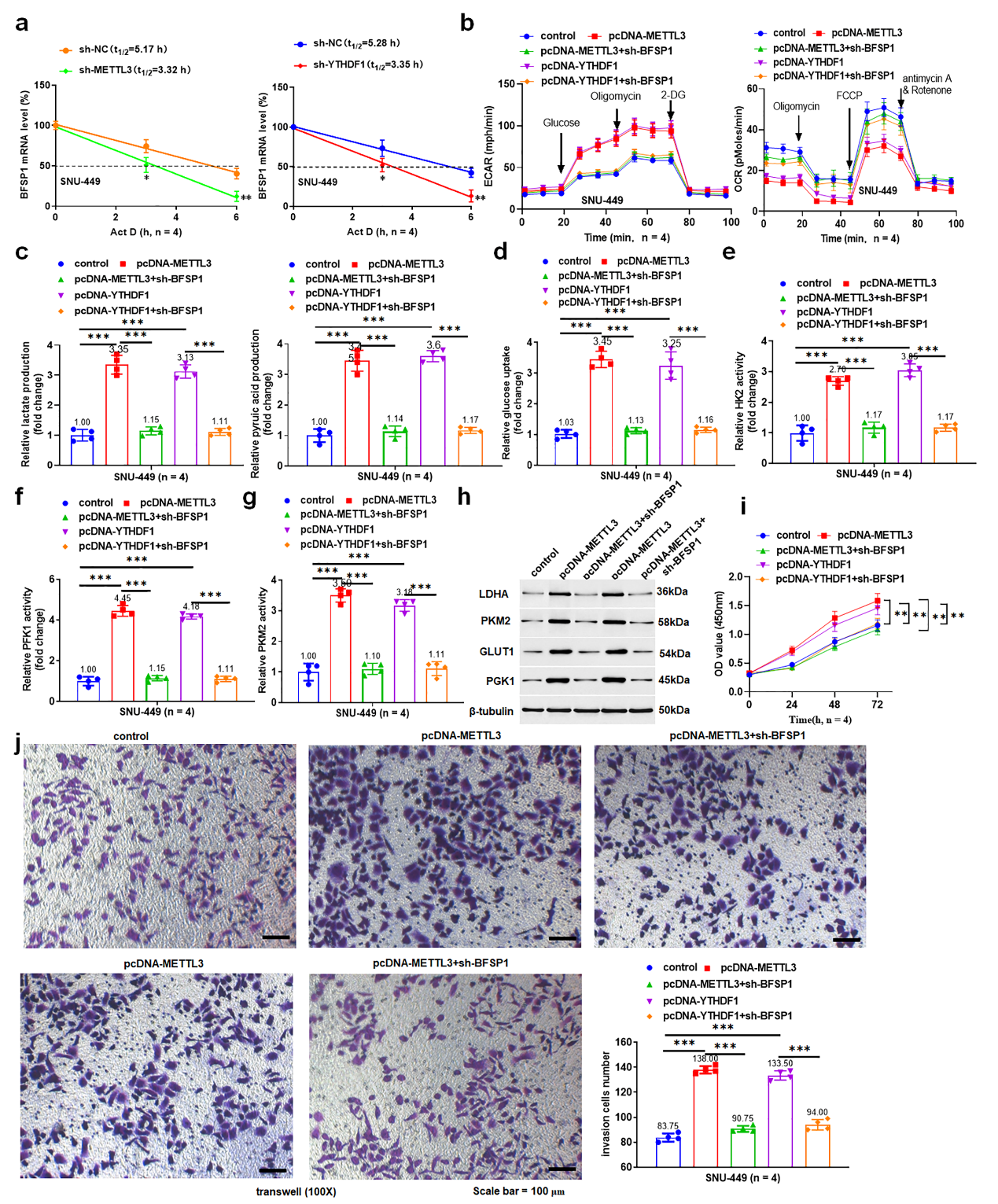


**Fig. S3 METTL3 mediated m6A modification of BFSP1 mRNA enhances BFSP1 stability and promotes aerobic glycolysis and invasion of SNU-449 cells in a YTHDF1 dependent manner**

**a.** The stability of BFSP1 mRNA (n = 4). **b.** The levels of ECAR and OCR in SNU-449 cells (n = 4). **c.** levels of lactic acid and pyruvate activity in SUN-449 cells (n = 4). **d.** Glucose uptake in SNU-449 cells (n = 4). **e-g.** The activities of HK2, PFK1, and PKM2 enzymes in SNU-449 cells (n = 4). **h.** The expression levels of glycolysis-related proteins in SNU-449 cells (n = 4). **i.** The viability of SNU-449 cells (n = 4). **j.** The invasion ability of SNU-449 cells (n = 4). ***P* < 0.01, ****P* < 0.001.


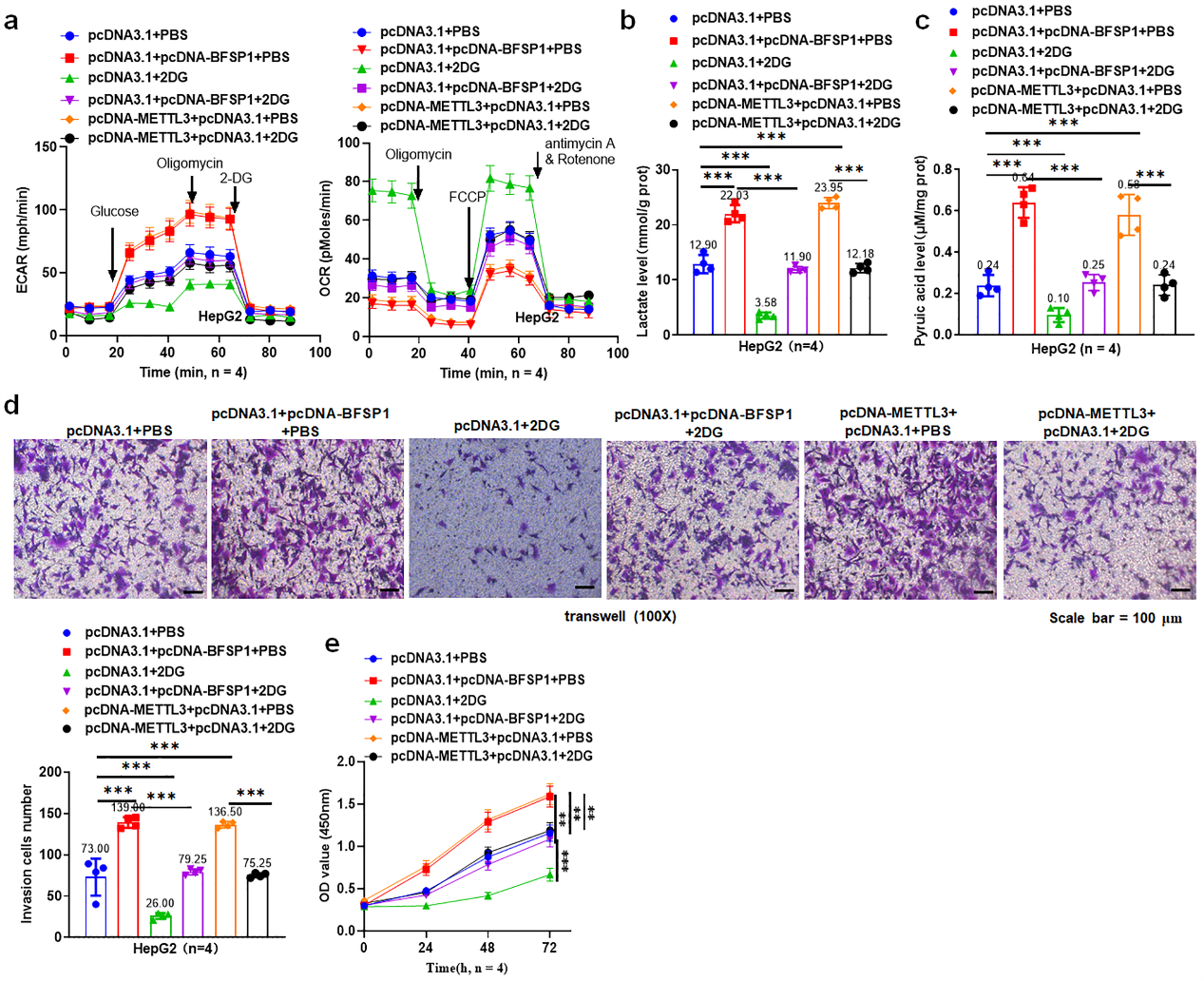


**Fig. S4 METTL3 mediated m6A modification of BFSP1 mRNA induces the viability and invasion of** **HepG2 cells by activating aerobic glycolysis**

**a.** The levels of ECAR and OCR in HepG2 cells (n = 4). **b.** The level of pyruvate in HepG2 cells (n = 4). **c.** The level of lactic acid in HepG2 cells (n = 4). **d.** The invasion ability of HepG2 cells (n = 4). **e.** The viability of HepG2 cells (n = 4). ***P* < 0.01, ****P* < 0.001.

**
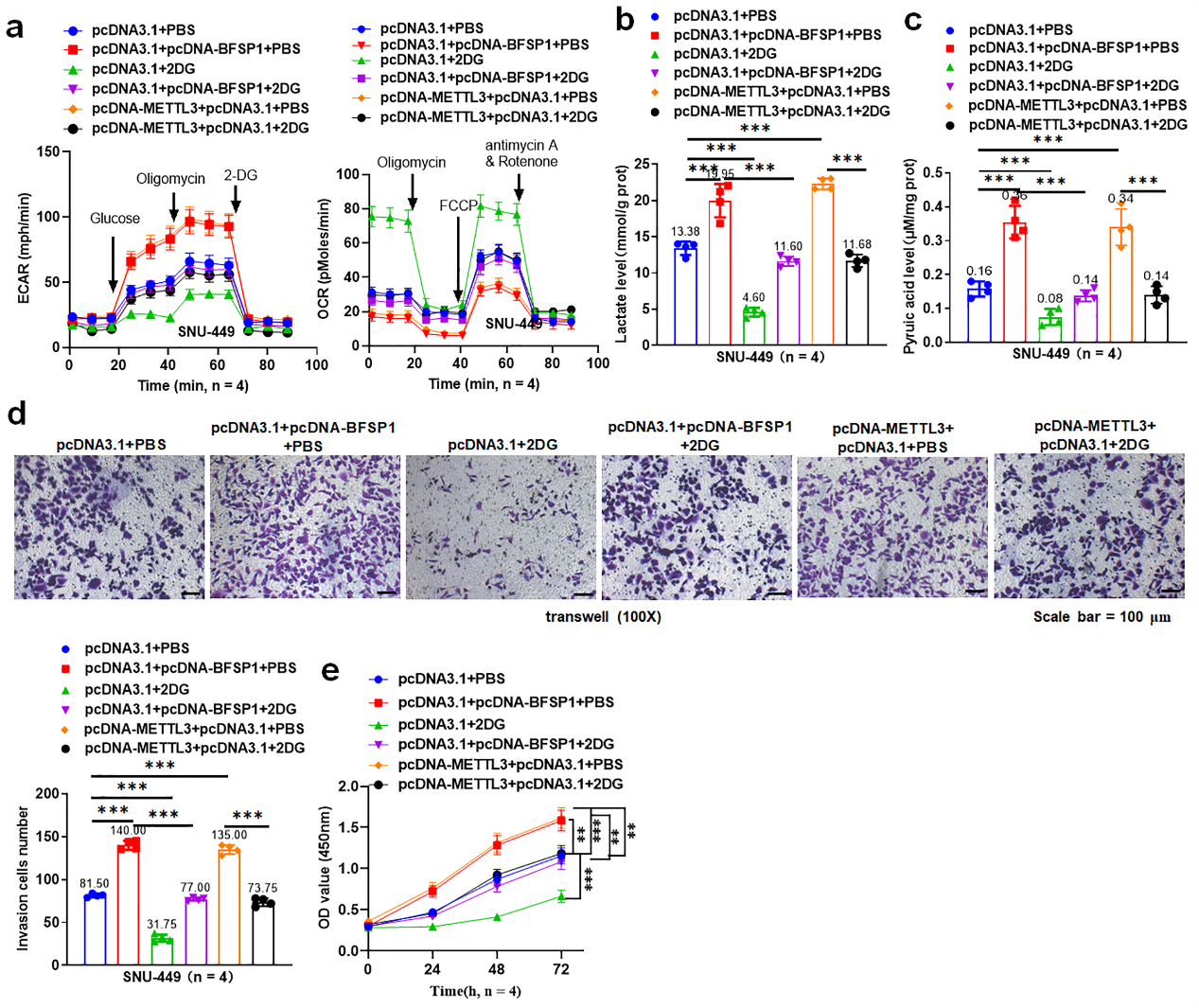
**

**Fig. S5 METTL3 mediated m6A modification of BFSP1 mRNA induces the viability and invasion of** **SNU-449 cells by activating aerobic glycolysis**

**a.** The levels of ECAR and OCR in SNU-449 cells (n = 4). **b.** The level of pyruvate in SNU-449 cells (n = 4). **c.** The level of lactic acid in SNU-449 cells (n = 4). **d.** The invasion ability of SNU-449 cells (n = 4). **e.** The viability of SNU-449 cells (n = 4). ***P* < 0.01, ****P* < 0.001.

**Supplementary Table**

Supplementary Table 1 qRT-PCR primer sequences

| Gene | sequence | |
| --- | --- | --- |
| BFSP1 | Forward Primer | 5'-CCATGCTGGAGAAAGTATGTATGAA-3' |
|  | Reverse Primer | 5'-GATGCTGGAGGAGTGGTGTG-3' |
| TMOD4 | Forward Primer | 5'-CCTACTGACACAGGGGAGGT-3' |
|  | Reverse Primer | 5'-CTGGCAGGAGCATGTTCTCA-3' |
| METTL3 | Forward Primer | 5'-TCAGCATCGGAACCAGCAAA-3' |
|  | Reverse Primer | 5'-TGGGGATTTCCTTTGACACCA-3' |
| YTHDF1 | Forward Primer | 5'-CGTGGACACCCAGAGAACAA-3' |
|  | Reverse Primer | 5'-TGCCCAAAAACAGCATCGTG-3' |
| β-actin | Forward Primer | 5'-TCGTGCGTGACATTAAGGAG-3' |
|  | Reverse Primer | 5'-GTCAGGCAGCTCGTAGCTCT-3' |

Supplementary Table 2 The information of antibodies

| Antibody | Catalog Number | Dilution ratio | Brand | Country |
| --- | --- | --- | --- | --- |
| anti-BFSP1 (WB) | ab126235 | 1:200 | Abcam | UK |
| anti-TMOD4 (WB) | ab240976 | 1:200 | Abcam | UK |
| anti-METTL3 (WB) | ab195352 | 1:200, | Abcam | UK |
| anti-YTHDF1 (WB) | ab220162 | 1:200 | Abcam | UK |
| anti-LDHA (WB) | ab101562 | 1:200 | Abcam | UK |
| anti-GLUT1 (WB) | ab115730 | 1:500 | Abcam | UK |
| anti-PGK1 (WB) | ab199438 | 1:400 | Abcam | UK |
| anti-PKM2 (WB) | ab150377 | 1:500 | Abcam | UK |
| anti-β-actin (WB) | ab124964 | 1:500 | Abcam | UK |
| anti-β-tubulin (WB) | ab78078 | 1:400 | Abcam | UK |
| IgG H&L (HRP) (WB) | ab6721 | 1:400 | Abcam | UK |
| anti-BFSP1 (IF) | ab251869 | 10 μg/mL | Abcam | UK |
| anti-TMOD4 (IF) | ab155244 | 1:50 | Abcam | UK |
| IgG (IF) | ab150077 | 1:50 | Abcam | UK |
| anti-TMOD4 (IHC) | ab251869 | 1:500 | Abcam | UK |
| anti-TMOD4 (IHC) | ab308621 | 1:200 | Abcam | UK |
| IgG (IHC) | ab672 | 1:200 | Abcam | UK |

**Raw date**

Figure1e BFSP1 Figure1eβ-actin Figure2b BFSP1


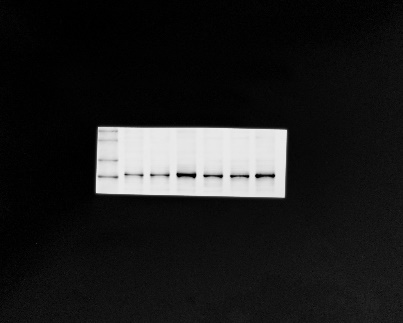

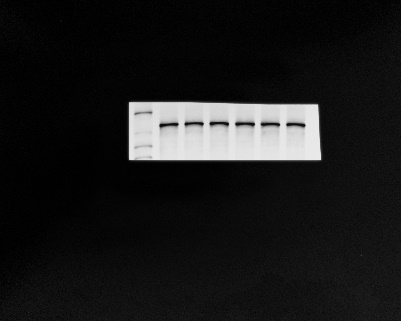

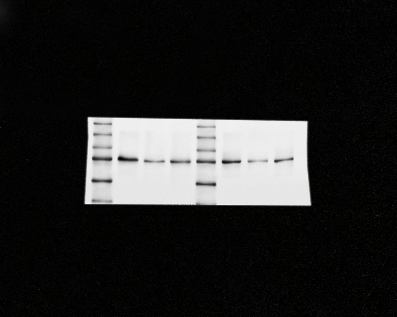


Figure2bβ-actin Figure2iβ-tubulin-1 Figure2i GLUT1-1


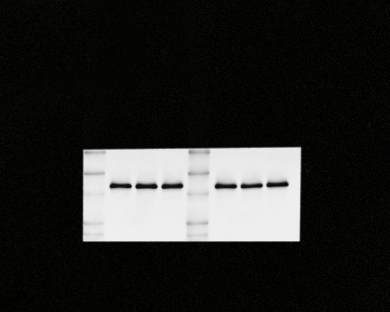

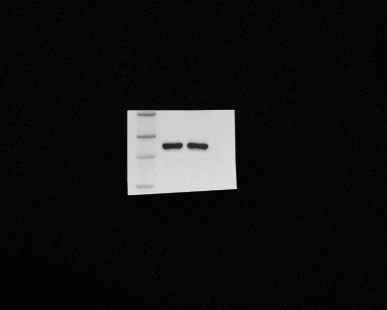

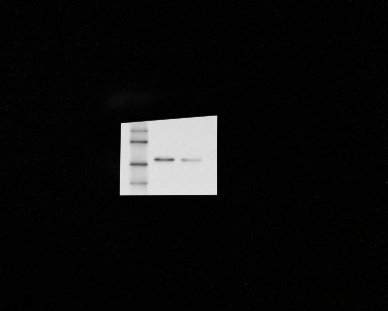


Figure2i LDHA-1 Figure2i PGK1-1 Figure2i PKM2-1


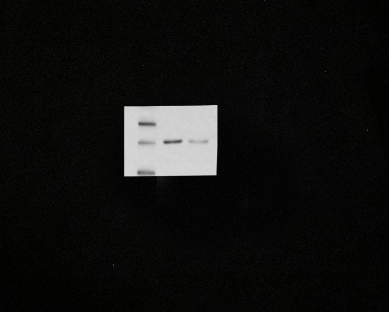

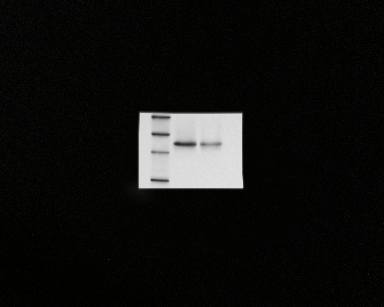

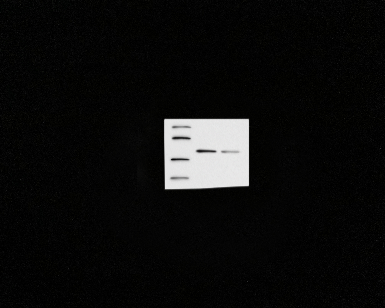


Figure2iβ-tubulin-2 Figure2i GLUT1-2 Figure2i LDHA-2


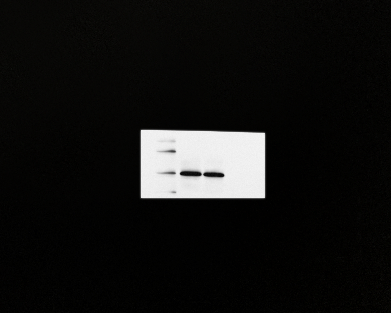

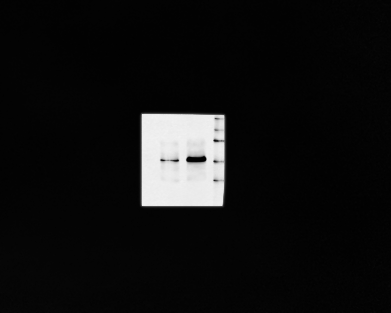

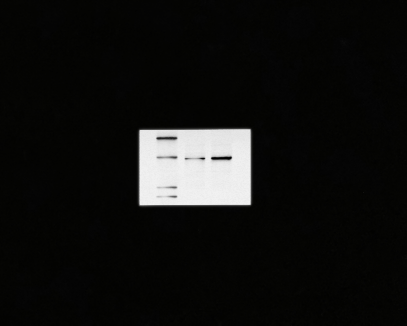


Figure2i PGK1-1 Figure2i PKM2-1


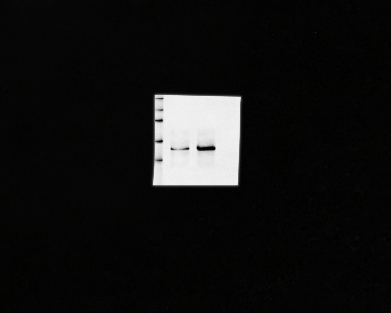

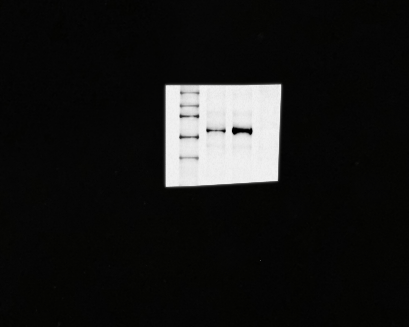


Figure3f TMOD4 Figure3fβ-actin Fig.3b-Input(anti-BFSP1-BFSP1)


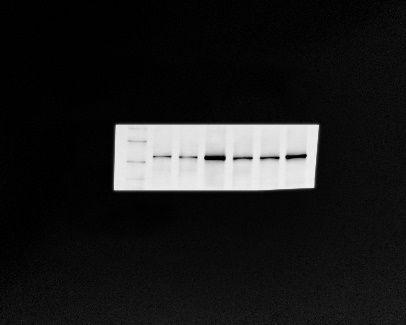

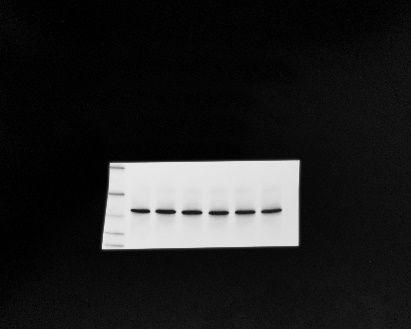

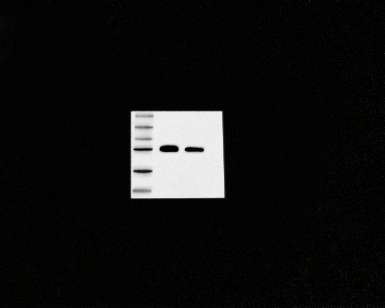


Fig.3b-Input(anti-BFSP1-TMOD4) Fig.3b-Input(anti-TMOD4-BFSP1) Fig.3b -Input(anti-TMOD4-GAPDH)


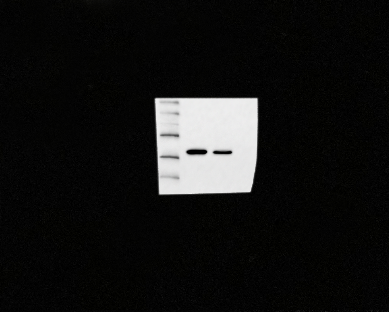

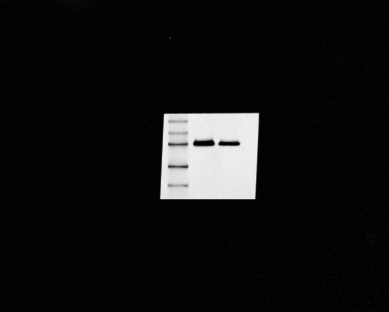

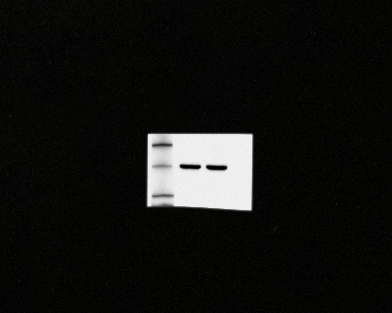


Fig.3b -Input(anti-TMOD4-TMOD4) Fig.3b -IP(anti-BFSP1-BFSP1) Fig.3b-IP(anti-BFSP1- GAPDH)


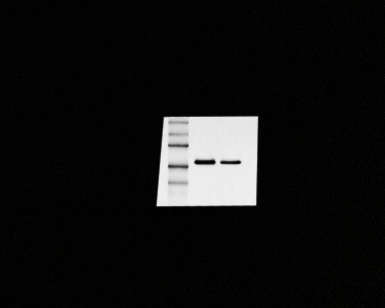

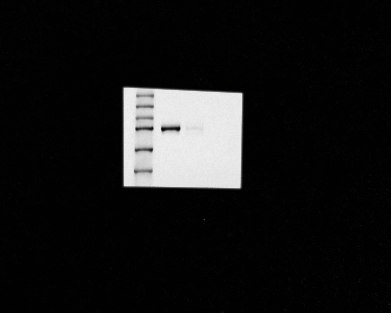

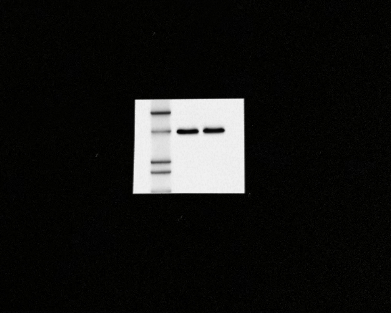


Fig.3b IP(anti-BFSP1-TMOD4) Fig.3b IP(anti-TMOD4-BFSP1) Fig.3b IP(anti-TMOD4-TMOD4)


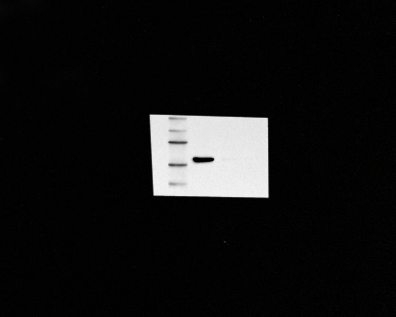

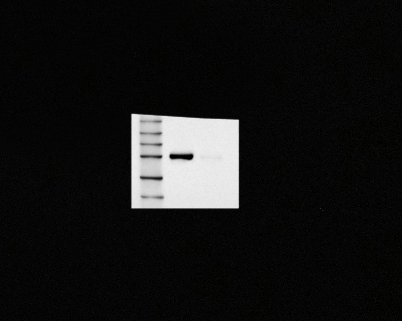

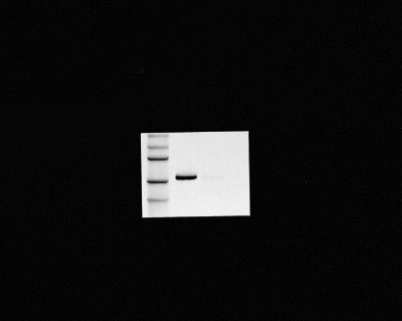


Figure3h TMOD4-1 Figure3hβ-actin-1 Figure3h BFSP1-1


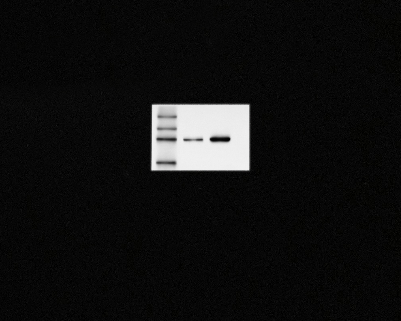

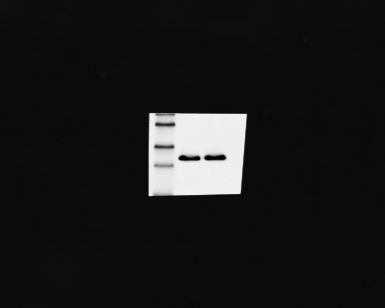

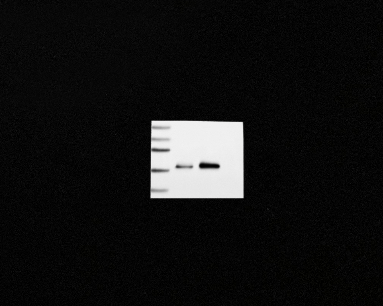


Figure3hβ-actin-2 Figure3h TMOD4-2 Figure3hβ-actin-3


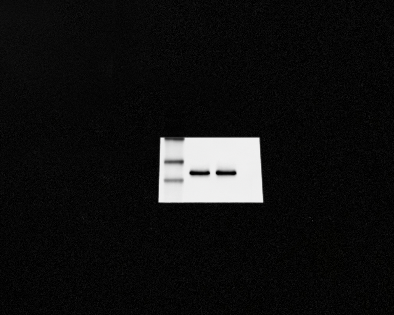

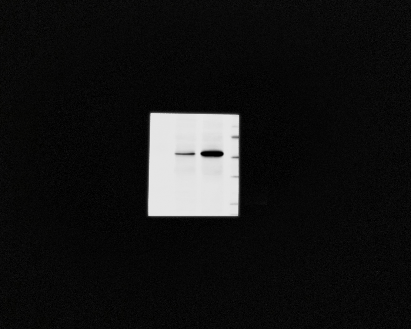

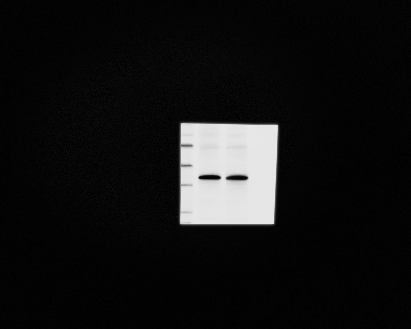


Figure3h BFSP1-2 Figure3hβ-actin-4 Figure3c GST -BFSP1


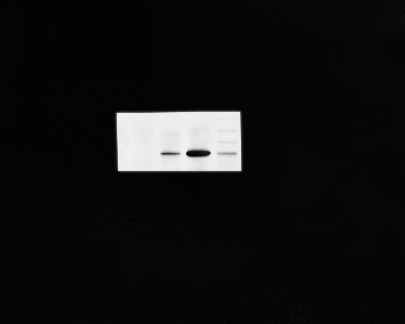

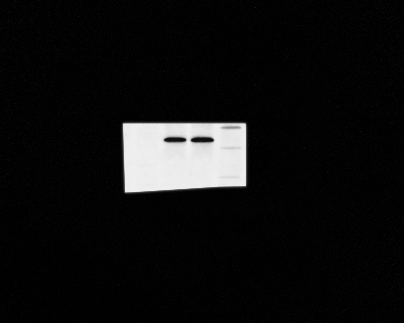

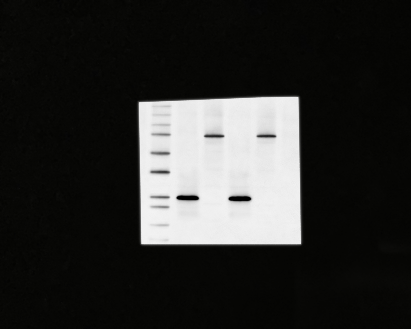


Figure3c His -TMOD4 Figure4 GLUT1 Figure4 LDHA


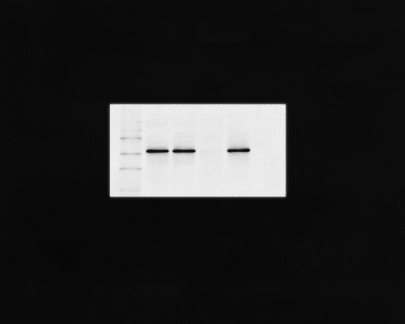

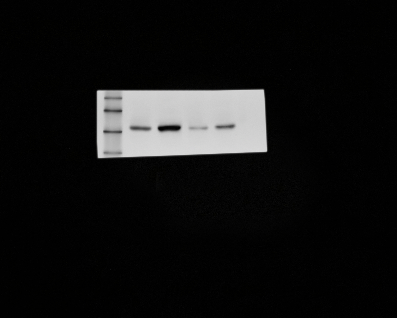

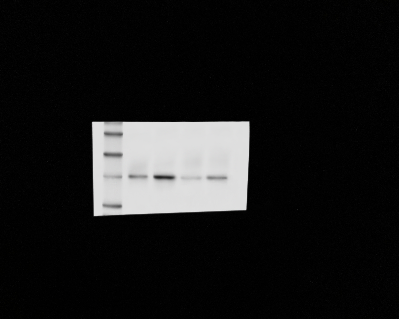


Figure4 PGK1 Figure4 PKM2 Figure4β-tubulin


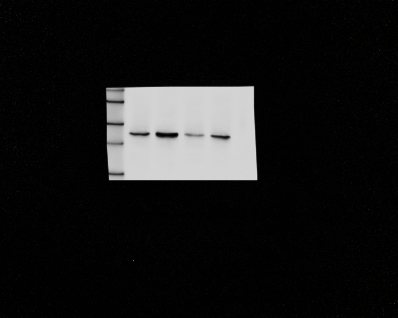

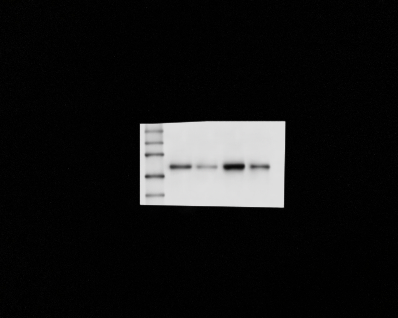

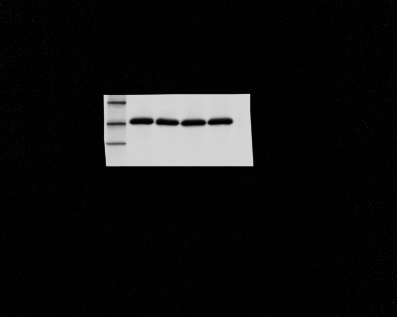


Figure5 METTL3 Figure5 β-actin Figure6e YTHDF1


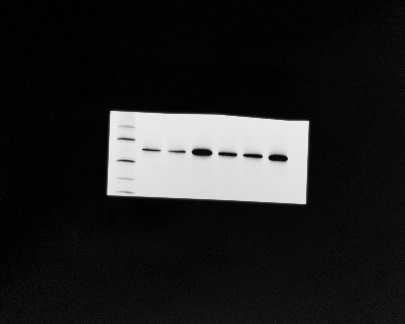

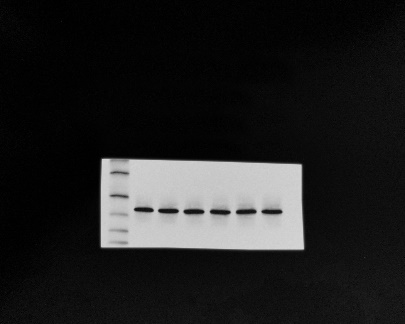

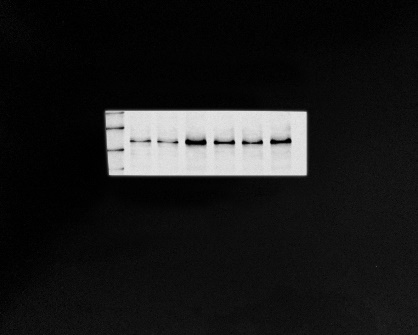


Figure6eβ-actin-1 Figure6g BFSP1 Figure6gβ-actin-2


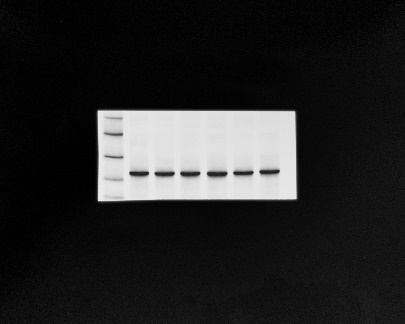

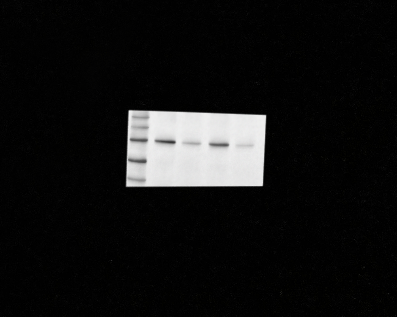

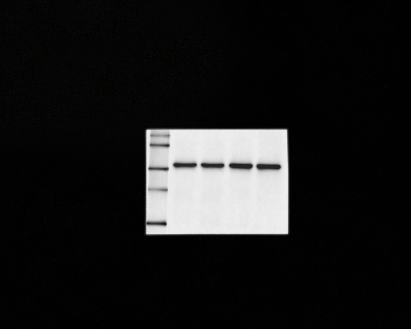


Figure7 GLUT1 Figure7 LDHA Figure7 PGK1


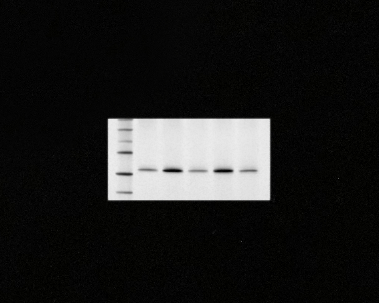

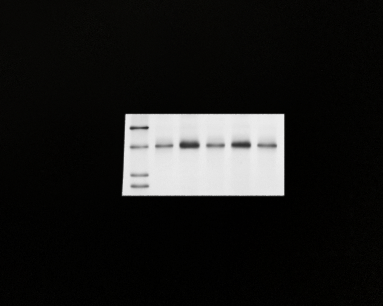

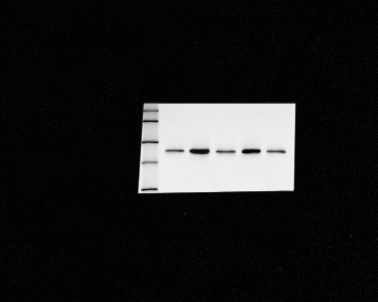


Figure7 PKM2 Figure7β-tubulin Figure8 β-actin


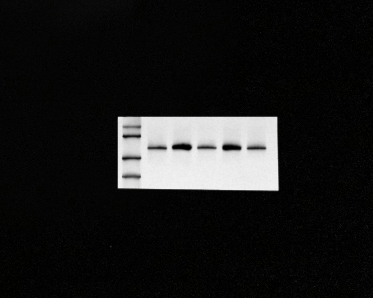

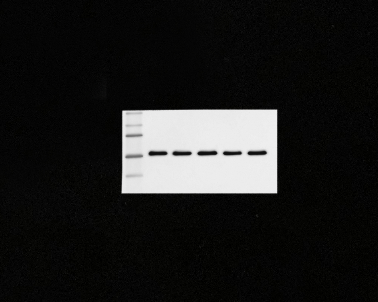

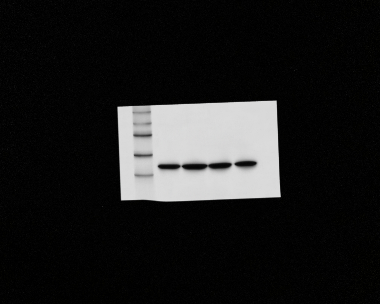


Figure8 BFSP1 Figure8 METTL3 Figure8 TMOD4


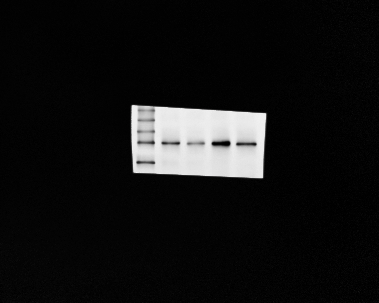

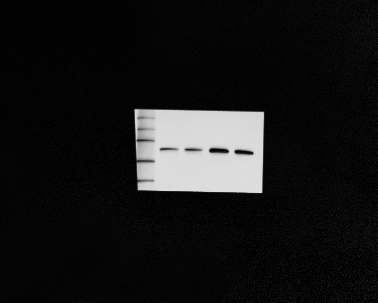

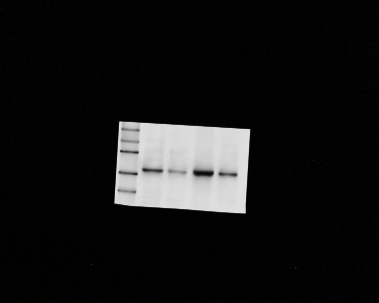


FigureS1β-tubulin Figure S1 GLUT1 Figure S1 LDHA


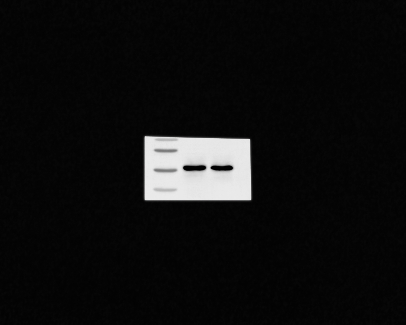

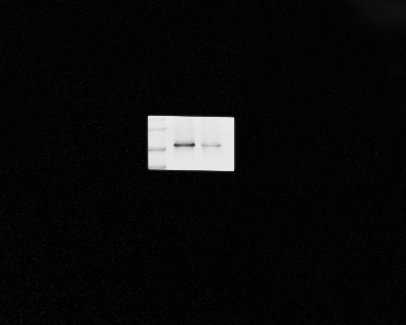

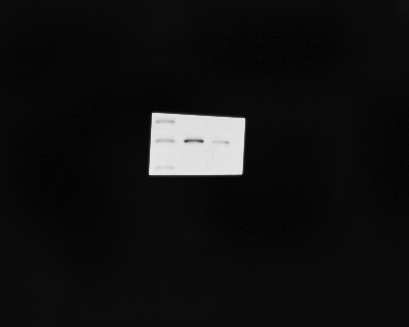


Figure S1 PGK1 Figure S1 PKM2 FigureS2β-tubulin


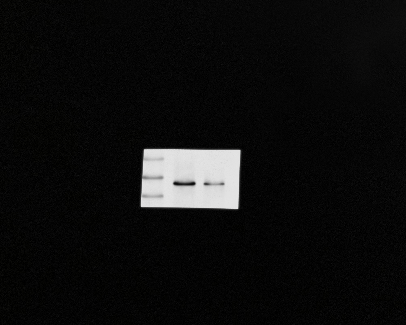

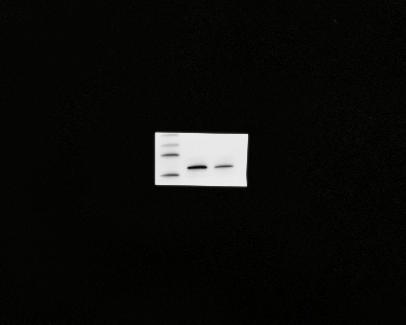

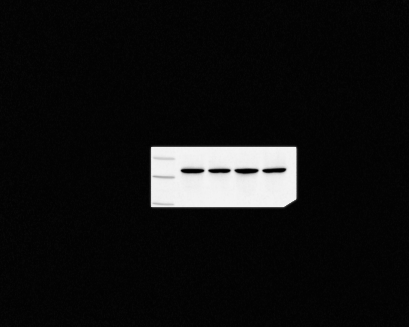


Figure S2 GLUT1 Figure S2 LDHA Figure S2 PGK1


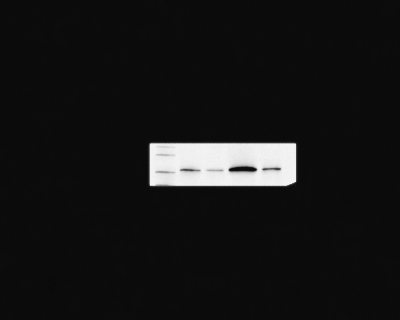

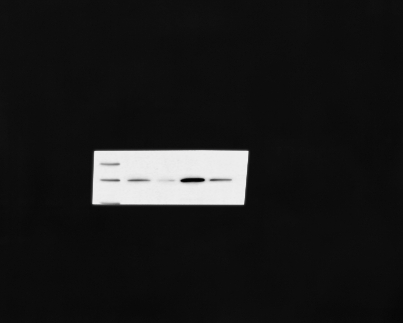

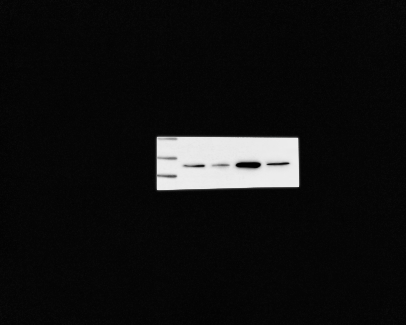


Figure S2 PKM2 FigureS3β-tubulin Figure S3 GLUT1


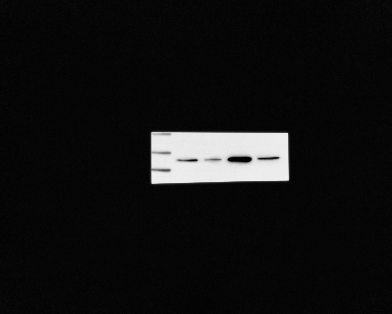

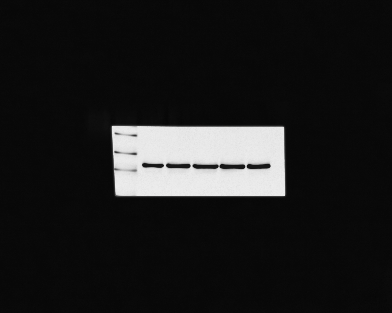

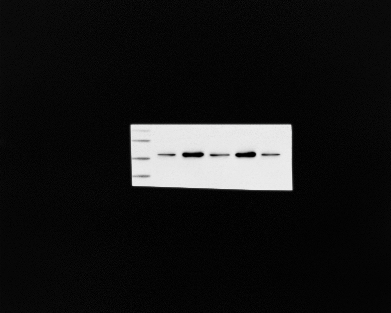


Figure S3 LDHA Figure S3 PGK1 Figure S3 PKM2


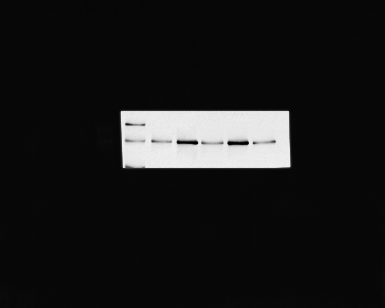

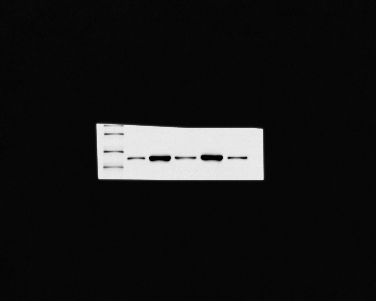

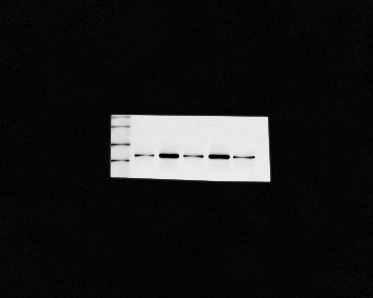

Supplement: Supplementary file 1 — Supplementary Material 1. [file 43556_2025_256_MOESM1_ESM.docx]
